# Characterization of key aroma compounds and regulation mechanism of aroma formation in local Binzi (*Malus pumila* × *Malus asiatica*) fruit

**DOI:** 10.1186/s12870-022-03896-z

**Published:** 2022-11-15

**Authors:** Qinghua Wang, Fan Gao, Xuexue Chen, Wenjiang Wu, Lei Wang, Jiangli Shi, Yun Huang, Yuanyue Shen, Guoliang Wu, Jiaxuan Guo

**Affiliations:** 1 College of Forestry, Henan Agricultural University, 450002 Zhengzhou, China; 2 Beijing Key Laboratory for Agricultural Application and New Technique, Beijing University of Agriculture, 102206 Beijing, China; 3 College of Horticulture, Henan Agricultural University, 450002 Zhengzhou, China; 4 College of Agronomy, Henan Agricultural University, 450002 Zhengzhou, China

**Keywords:** *Malus*, Binzi, Fruit aroma, Post-harvest, Gene expression

## Abstract

**Background:**

Volatile components are important secondary metabolites essential to fruit aroma quality, thus, in the past decades many studies have been extensively performed in clarifying fruit aroma formation. However, aroma components and biosynthesis in the fruit of Binzi (*Malus pumila* × *Malus asiatica*), an old local species with attractive aroma remain unknown.

**Results:**

We investigated two Binzi cultivars, ‘Xiangbinzi’ (here named high-fragrant Binzi, ‘HFBZ’) and ‘Hulabin’ (here named low-fragrant Binzi, ‘LFBZ’) by monitoring the variation of volatiles and their precursors by Gas Chromatography–Mass Spectrometer (GC–MS), as well as their related genes by RNA-seq during post-harvest ripening. We firstly confirmed that ‘HFBZ’ and ‘LFBZ’ fruit showed respiratory climacteric by detecting respiratory rate and ethylene emission during post-harvest; found that esters were the major aroma components in ‘HFBZ’ fruit, and hexyl 2-methylbutyrate was responsible for the ‘fruity’ note and most potent aroma component, followed by ethyl acetate, ethyl butanoate, (*E*)-2-hexenal, and 1-hexanol. Regarding aroma synthesis, fatty acid metabolism seemed to be more important than amino acid metabolism for aroma synthesis in ‘HFBZ’ fruit. Based on RNA**-**seq and quantitative reverse transcription PCR (RT-qPCR), *LOX2a*, *LOX5a*, *ADH1*, and *AAT1* genes are pointed to the LOX pathway, which may play a vital role in the aroma formation of ‘HFBZ’ fruit.

**Conclusion:**

Our study firstly investigated the aroma components and related genes of Binzi fruit, and provided an insight into the fragrant nature of *Malus* species.

**Supplementary Information:**

The online version contains supplementary material available at 10.1186/s12870-022-03896-z.

## Background

Binzi, belonging to the Rosaceae family, is an old local species in Beijing from a natural hybrid of paradise apple (*Malus* ⋅ *pumila*) and Chinese pearleaf crabapple (*Malus* ⋅ *asiatica*) [1]. It is known that Binzi included ‘Xiangbinzi’ (here named high-fragrant Binzi, ‘HFBZ’) and ‘Hulabin’ (here named low-fragrant Binzi, ‘LFBZ’) cultivars. The harvested ‘HFBZ’ fruit appears attractive color, moderate size, and full aroma as a ‘fruity’ note [2]. Contrary to the ‘HFBZ’ fruit, the ‘LFBZ’ fruit appears a rather faint aroma [3]. The Binzi trees were widely cultivated in Beijing before 1990s. Hereafter, with the rise of the ‘Red Fuji’ apple in China, the Binzi trees were gradually cut down for ‘Fuji’ planting. In recent years, in order to save the valuable species, the Beijing government has initiated a project for the protection, utilization, and restoration of this local species. However, aroma compounds essential to the fragrant quality of Binzi fruit remain unknown. This evoked us to explore aroma compounds in Binzi fruit.

Aroma is an important flavor characteristic for fruit quality, attractive to producers and consumers [4]. More than 1,000 olfactory genes are related to odor sensing, and the aroma components affect our perception of odor [5, 6]. In recent years, nearly 2,000 volatile aroma components have been identified from different plant species, including apple (*Malus* ⋅ *domestica*), pear (*Pyrus* ⋅ *ussuriensis*), and strawberry (*Fragaria* ⋅ *ananassa*), and are divided into esters, aldehydes, alcohols, ketones, olefins, and acids [7–10]. In apple, more than 300 aroma components have been identified [11]. About 51 aroma components are identified in ‘Honeycrisp’ apple, and esters are most prominent [9]; similarly, 53 aroma compounds in ‘Fuji’ apple include 27 esters, 12 aldehydes, and 5 alcohols [12]. Aldehydes are mainly present in the immature apple fruit from cellular disruption, while esters are generated from ripe fruit due to cell walls and membrane more permeable [13, 14].

The secondary metabolites related to aroma components are mainly generated from fatty acid, carbohydrate, and amino acid metabolism [15]. In the fatty acid metabolism pathway, straight-chain aldehydes, alcohols, and esters are mainly synthesized from the linoleic acid (C_18:2_) and linolenic acid (C_18:3_) [16]. In addition, terpenoids and amino acids are important and contribute to many of the odor active constituents [7]. Branched amino acids, including isoleucine (Ile), leucine (Leu), and valine (Val), are precursors of branched-chain esters in apple fruit [17]. Besides these, a recent study showed that the citramalate pathway is also involved in ester formation during fruit ripening [18].

Lipoxygenase (LOX) pathway is one of the main fatty acid metabolisms involved in volatile synthesis [19]. To date, the volatile aroma genes encoding LOX, hydroperoxide lyase (HPL), alcohol dehydrogenase (ADH), and alcohol acyltransferase (AAT) have been characterized in apple, papaya (*Carica* ⋅ *papaya*), cherry tomato (*Solanum* ⋅ *lycopersicum*), and pear [10, 20–22]. LOX plays a vital role in fruit ripening and fresh fruit aroma can be influenced by LOX activity [23]. LOX enzymes are also classified into 9-LOX or 13-LOX according to the carbon targeted for deoxygenation in the unsaturated fatty acid [7]. In ‘Nanguo’ pear, the expression of *PuLOX3*, *PuADH3*, and *PuAAT* genes contributed to the formation of the total esters during post-harvest ripening [10]. Cao et al. found that the transcript levels of *PpAAT1* showed a significant positive correlation with the content of esters (*p* < 0.001), and over-expression of *PpAAT1* increased production of volatile esters [24]. However, whether these candidate genes participate in volatiles of Binzi fruit remains to be determined.

Although considerable progress has been made in clarifying aroma components and biosynthesis in several fruits, the aroma of Binzi fruit remains unknown. This study aimed to identify the composition and concentration of volatile components and elucidate the mechanism of aroma formation in Binzi fruit, by using the comprehensive comparison of metabolite accumulation and transcriptome profiles between ‘HFBZ’ and ‘LFBZ’ fruit. In addition, RT-qPCR was used to evaluate the potential function of candidate genes related to aroma components after harvest. Our findings will contribute to the understanding of the mechanism of aroma formation and provide valuable information for Binzi breeding.

## Results

### Physiological characteristics of Binzi fruit after harvest

**Fig. 1:**
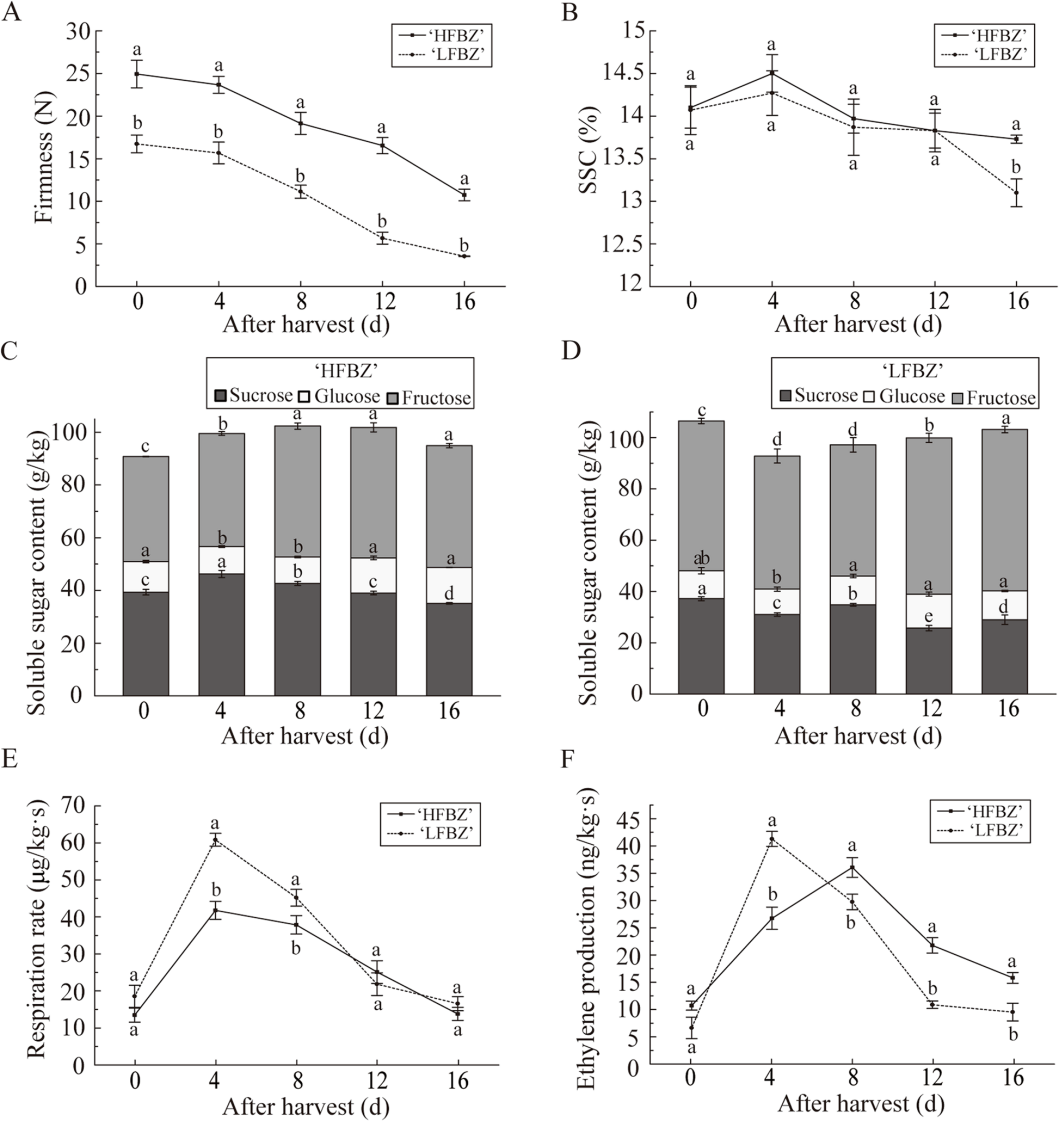
Physiological characteristics of ‘HFBZ’ and ‘LFBZ’ fruit after harvest. **a** Firmness in ‘HFBZ’ and ‘LFBZ’ fruit after harvest. **b** Soluble solid content (SSC) in ‘HFBZ’ and ‘LFBZ’ fruit after harvest. **c** Sucrose, glucose, and fructose contents in ‘HFBZ’ fruit after harvest. **d** Sucrose, glucose, and fructose contents in ‘LFBZ’ fruit after harvest. **e** Respiration rate in ‘HFBZ’ and ‘LFBZ’ fruit after harvest. **f** Ethylene production in ‘HFBZ’ and ‘LFBZ’ fruit after harvest. Values represent means of the three replicates; bars represent standard deviation of the three replicates. Lowercase letters, a, b, c, and d represent significant differences according to the independent sample t-test (*p* < 0.05) for each sampling time point

One of the striking features of ‘HFBZ’ fruit is the bright red color with high anthocyanin accumulation in peels (Supplementary Fig. S1). Firmness of ‘HFBZ’ and ‘LFBZ’ fruit declined synchronously after harvest, and higher firmness was observed in ‘HFBZ’ fruit (Fig. 1a). There was no significant difference in SSC of the ‘HFBZ’ and ‘LFBZ’ fruit except at day 16, and the SSC of the two cultivars reached the peak at 4 d after harvest (Fig. 1b). Saccharides are an important factor influencing fruit flavor, and total contents of sucrose, glucose, and fructose showed no significant variation in the two cultivars after harvest (Fig. 1c, d, and Supplementary Fig. S2). The respiratory rates of ‘HFBZ’ and ‘LFBZ’ fruit showed similar trends: initially increasing, reaching a peak rate at day 4, and then decreasing; overall, it was higher in the ‘LFBZ’ fruit than in the ‘HFBZ’ fruit at day 4 and day 8 after harvest (Fig. 1e). In addition, the ‘HFBZ’ fruit reached peak of ethylene production at day 8 after harvest, while the ‘LFBZ’ fruit early reached the peak at day 4 (Fig. 1f). Taken together, apple-like Binzi fruit show typical climacteric during post-harvest ripening, and the peaks of respiratory climacteric (PRC) in ‘HFBZ’ and ‘LFBZ’ fruit are at day 4 after harvest.

### Variation in the components and contents of volatiles in Binzi fruit after harvest

**Fig. 2:**
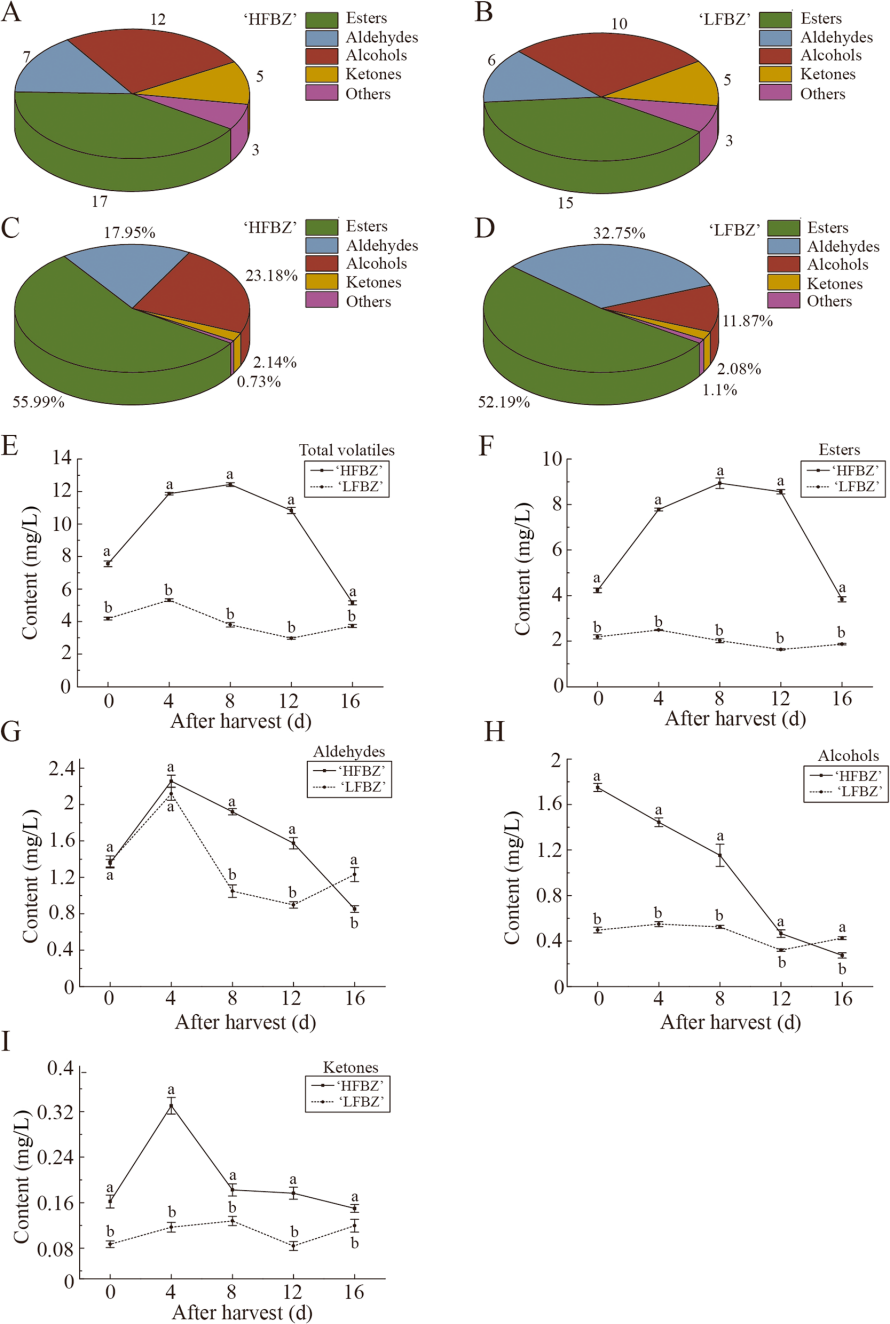
Variation in volatile aroma compounds of ‘HFBZ’ and ‘LFBZ’ fruit after harvest. **a** Components of volatiles in ‘HFBZ’ fruit at harvest day (day 0). **b** Components of volatiles in ‘LFBZ’ fruit at harvest day (day 0). **c** Proportion of different volatiles in ‘HFBZ’ fruit at harvest day (day 0). **d** Proportion of different volatiles in ‘LFBZ’ fruit at harvest day (day 0). **e** Total volatiles in ‘HFBZ’ and ‘LFBZ’ fruit after harvest. **f** Esters in ‘HFBZ’ and ‘LFBZ’ fruit after harvest. **g** Aldehydes in ‘HFBZ’ and ‘LFBZ’ fruit after harvest. **h** Alcohols in ‘HFBZ’ and ‘LFBZ’ fruit after harvest. **i** Ketones in ‘HFBZ’ and ‘LFBZ’ fruit after harvest. Values represent means of the three replicates; bars represent standard deviation of the three replicates. Lowercase letters, a and b, represent significant differences according to the independent sample t-test (*p* < 0.05) for each sampling time point

We compared the volatile components of the two Binzi cultivars, ‘HFBZ’ and ‘LFBZ’ fruit at harvest day (day 0). A total of 44 volatile components were detected in the ‘HFBZ’ fruit (Fig. 2a and Supplementary Table S1). Esters were the most abundant (17), followed by alcohols (12), aldehydes (7), ketones (5), benzenoid (1), acids (1), and sesquiterpene (1). A total of 39 volatile components were identified in ‘LFBZ’ fruit, including 15 esters, 10 alcohols, 6 aldehydes, 5 ketones, 1 benzenoid, 1 acid, and 1 sesquiterpene (Fig. 2b and Supplementary Table S1). Variety of fruit volatile components were generally similar between the ‘HFBZ’ and ‘LFBZ’ fruit. Furthermore, the proportion of esters in ‘HFBZ’ and ‘LFBZ’ fruit reached 56% and 52% at harvest day, respectively (Fig. 2c and d). The results corroborated esters were the main aroma components of the ‘HFBZ’ and ‘LFBZ’ fruit.

Next, we further quantitatively investigated the dynamic variation of volatiles contents in ‘HFBZ’ and ‘LFBZ’ fruit after harvest. The contents of total volatiles in ‘HFBZ’ fruit were always higher than those in the ‘LFBZ’ fruit, and increased to12.4 mg/L at day 8 (Fig. 2e). The esters of ‘HFBZ’ fruit gradually increased and reached a peak at day 8 and were remarkably higher than those in ‘LFBZ’ fruit (Fig. 2f). Regarding aldehydes, no significant difference was observed in ‘HFBZ’ and ‘LFBZ’ fruit before day 4, but then the aldehydes of ‘LFBZ’ fruit decreased more evidently (Fig. 2g). Interestingly, the alcohols of ‘HFBZ’ fruit continued to decline and finally even lower than those in ‘LFBZ’ fruit (Fig. 2h). Ketones in ‘HFBZ’ and ‘LFBZ’ fruit initially increased and then declined after harvest while the ketones were remarkably higher in ‘HFBZ’ fruit (Fig. 2i). Overall, it is inferred that it was the content of aroma components, rather than the variety, that was vital to the different odor in ‘HFBZ’ and ‘LFBZ’ fruit.

**Fig. 3:**
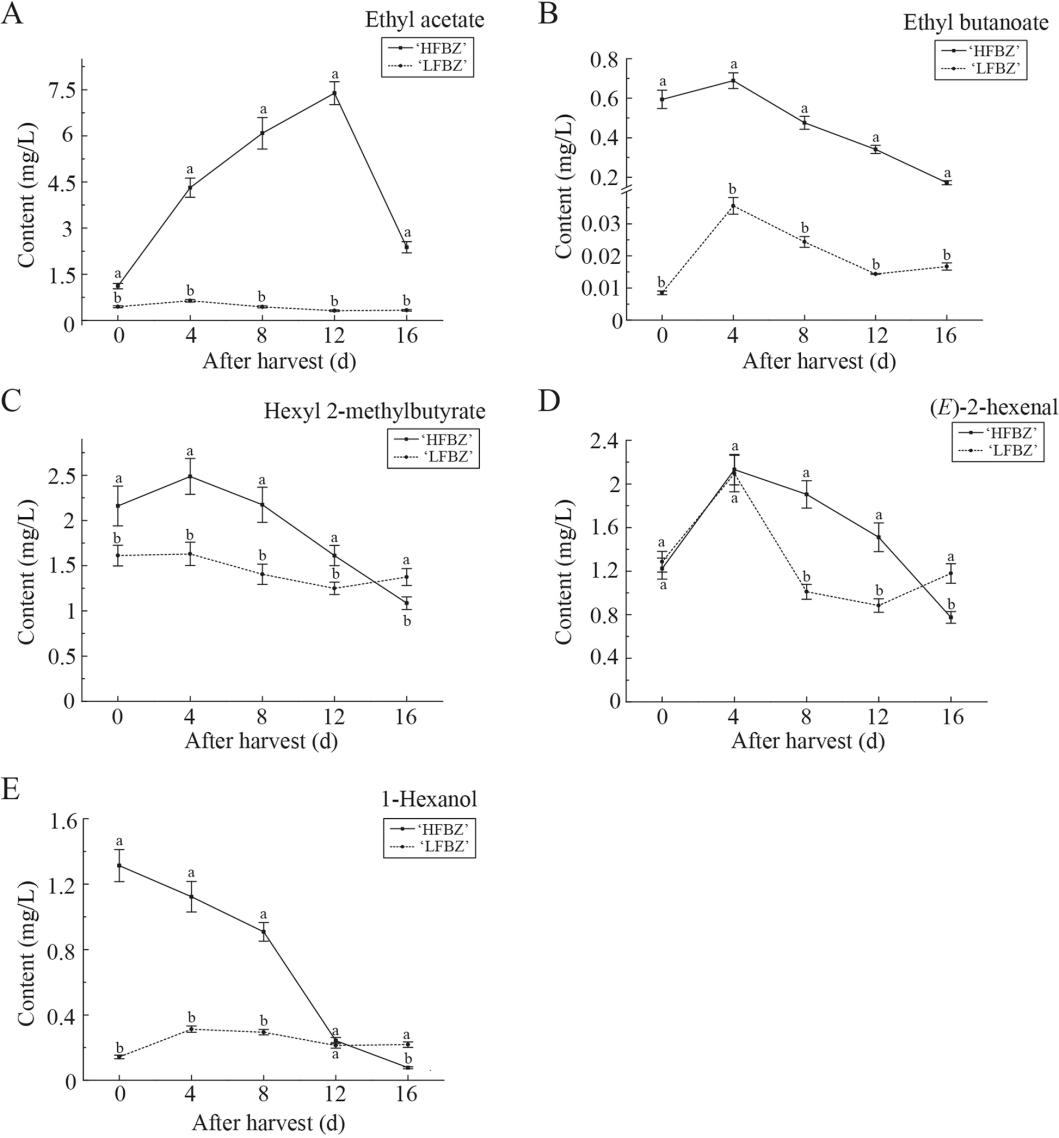
Variation of major aroma compounds in ‘HFBZ’ and ‘LFBZ’ fruit after harvest. **a** Ethyl acetate in ‘HFBZ’ and ‘LFBZ’ fruit after harvest. **b** Ethyl butanoate in ‘HFBZ’ and ‘LFBZ’ fruit after harvest. **c** Hexyl 2-methylbutyrate in ‘HFBZ’ and ‘LFBZ’ fruit after harvest. **d** (*E*)-2-hexenal in ‘HFBZ’ and ‘LFBZ’ fruit after harvest. **e** 1-hexanol in ‘HFBZ’ and ‘LFBZ’ fruit after harvest. Values represent means of the three replicates; bars represent standard deviation of the three replicates. Lowercase letters, a and b, represent significant differences according to the independent sample t-test (*p* < 0.05) for each sampling time point

In order to investigate which components contribute more to the aroma synthesis of the ‘HFBZ’ fruit, we screened out five major components from the ‘HFBZ’ fruit. As revealed in Supplementary Table S1, ethyl acetate, ethyl butanoate, hexyl 2-methylbutyrate, (*E*)-2-hexenal, and 1-hexanol mostly accumulated in ‘HFBZ’ fruit in higher concentrations than the other components. Additionally, the five aroma components contents were higher in ‘HFBZ’ fruit than those in ‘LFBZ’ fruit, especially the ethyl acetate, ethyl butanoate, and 1-hexanol (Fig. 3). The ethyl acetate in ‘HFBZ’ fruit rose gradually until reaching a peak at day 12, and then decreased (Fig. 3a). Ethyl butanoate, hexyl 2-methylbutyrate, and (*E*)-2-hexenal in ‘HFBZ’ fruit reached the peak at the PRC stage, then gradually declined, in consistent with the changes of respiratory rates (Fig. 3b and c). No significant difference of the (*E*)-2-hexenal content observed in ‘HFBZ’ and ‘LFBZ’ fruit before day 4; however, the (*E*)-2-hexenal in ‘LFBZ’ fruit then decreased rapidly, and was lower significantly than in ‘HFBZ’ fruit for the next time, except day16 (Fig. 3d). The 1-hexanol gradually decreased after postharvest (Fig. 3e). It seemed that high accumulation of ethyl acetate, ethyl butanoate, hexyl 2-methylbutyrate, (*E*)-2-hexenal, and 1-hexanol might be most responsible for higher aroma in ‘HFBZ’ fruit.

### Variation in free fatty acids and free amino acids contents of Binzi fruit after harvest

**Fig. 4:**
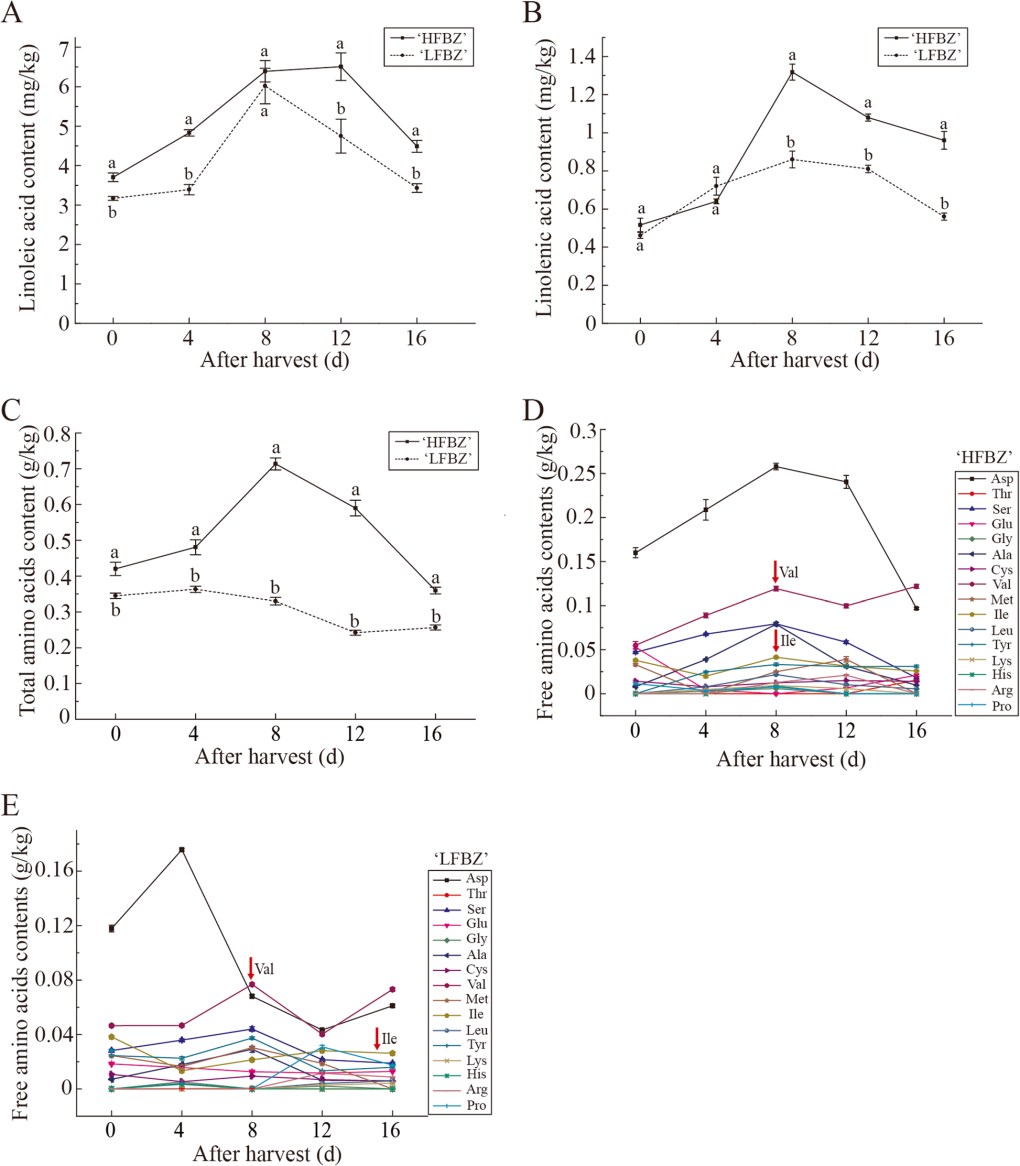
Variation in free fatty acids and free amino acids contents of Binzi fruit after harvest. **a** Variation in linoleic acid in ‘HFBZ’ and ‘LFBZ’ fruit after harvest. **b** Variation in linolenic acid in ‘HFBZ’ and ‘LFBZ’ fruit after harvest. **c** Variation in total amino acids in ‘HFBZ’ and ‘LFBZ’ fruit after harvest. **d** Variation in free amino acids in ‘HFBZ’ fruit after harvest. **e** Variation in free amino acids in ‘LFBZ’ fruit after harvest. Values represent means of three replicates; bars represent standard deviation of the three replicates. Lowercase letters, a and b, represent significant differences according to the independent sample t-test (*p* < 0.05) for each sampling time point

To investigate the fatty acids related to aroma components in ‘HFBZ’ fruit, linoleic acid and linolenic acid were detected in ‘HFBZ’ and ‘LFBZ’ fruit. Linoleic acid and linolenic acid had similar trends, initially rising greatly and then decreasing during post-harvest in both ‘HFBZ’ and ‘LFBZ’ fruit (Fig. 4a and b). Compared with ‘LFBZ’ fruit, the ‘HFBZ’ fruit accumulated more linoleic acid and linolenic acid. Additionally, the linolenic acid increased rapidly from day 4 and reached a peak at day 8, corresponding to the most accumulation of total volatiles and esters (Fig. 4b). These results suggested that the high accumulation of linoleic acid and linolenic acid during post-ripening may be responsible for high aroma in ‘HFBZ’ fruit.

Free amino acids had also been determined in ‘HFBZ’ and ‘LFBZ’ fruit. As shown in Fig. 4c, d and e, a total of 16 free amino acids were detected in ‘HFBZ’ and ‘LFBZ’ fruit, and their total content was higher in ‘HFBZ’ fruit. Only 10 free amino acids were detected in ‘HFBZ’ fruit at harvest day, while the number of amino acids increased to 14 at day 8 after harvest (Fig. 4d). Moreover, aspartic acid (Asp), valine (Val), alanine (Ala), isoleucine (Ile), and serine (Ser) were the main amino acids, accounting for 80% of the total free amino acids. Among the five major amino acids, the Val and Ile belongs to branched-chain amino acids, which are the precursors of branched-chain esters. However, no significant difference was found in Val and Ile contents between the ‘HFBZ’ and ‘LFBZ’ fruit (Fig. 4d and e), it seemed that the effect of branched-chain amino acids on the aroma difference between ‘HFBZ’ and ‘LFBZ’ fruit was not significant.

### Transcriptomic differences in the two Binzi cultivars after harvest

Based on the aroma variation trend of the ‘HFBZ’ and ‘LFBZ’ fruit after harvest, cDNA libraries from four time points (at day 0, 4, 8, and 12) of the two cultivars (‘HFBZ’ and ‘LFBZ’) were constructed for RNA-seq analysis. Approximately 6–7 Gb of clean bases were obtained. The Q30 base percentage was greater than 95.24% among each library (HF0, HF4, HF8, HF12, LF0, LF4, LF8, and LF12), and the average ‘G + C’ content of the above was approximately 47% (Supplementary Table S2).

**Fig. 5:**
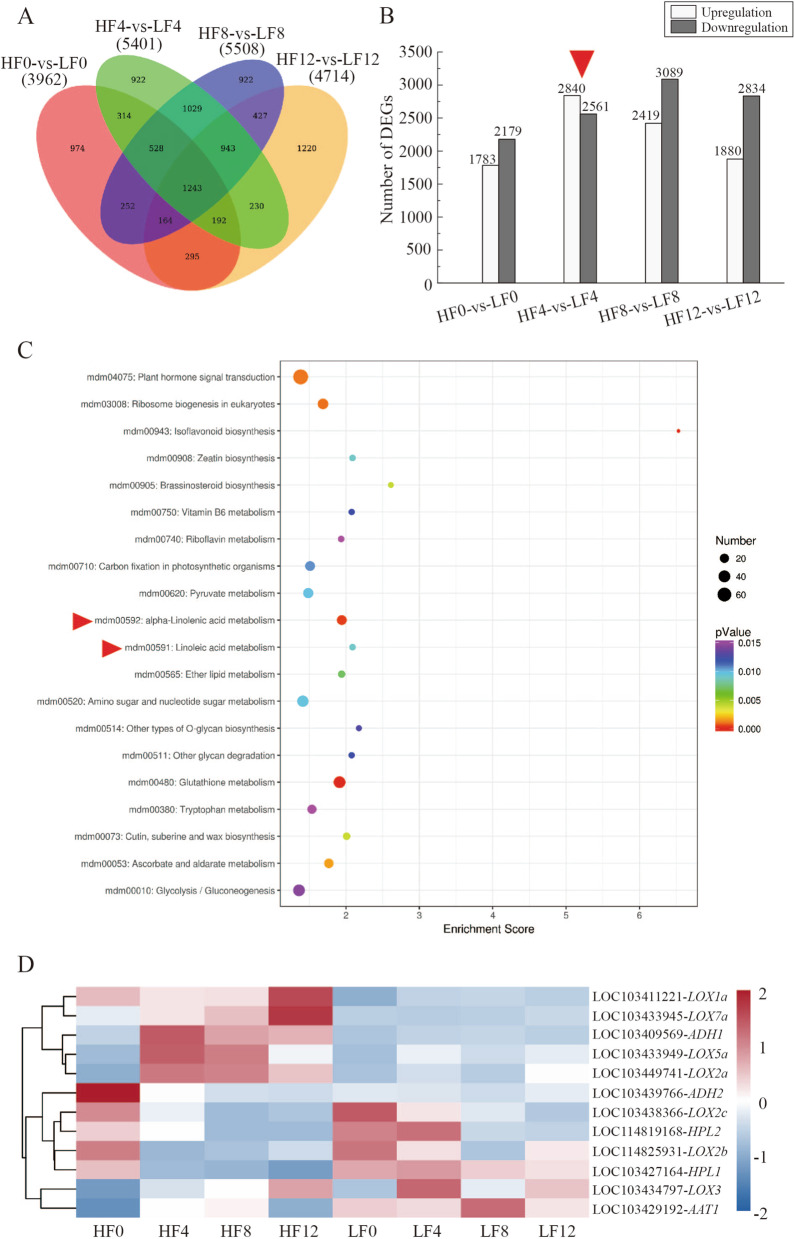
DEGs in ‘HFBZ’ and ‘LFBZ’ fruit after harvest were determined by RNA-seq. **a** Venn diagram for the different DEGs between library pairs. **b** The number of upregulated and downregulated DEGs between library pairs. **c** KEGG enrichment top 20 in HF4-vs-LF4. **d** Heat map and cluster analysis of the DEGs (*p* < 0.05 and foldchange > 2)

To explore the gene expression profiles of ‘HFBZ’ and ‘LFBZ’ fruit, the differentially expressed genes (DEGs) between each library pair (HF0-vs-LF0, HF4-vs-LF4, HF8-vs-LF8, and HF12-vs-LF12) were listed in Fig. 5. Comparing HF0 with LF0, HF4 with LF4, HF8 with LF8, and HF12 with LF12, 3,962, 5,401, 5,508, and 4,714 DEGs were found, respectively (Fig. 5a). Furthermore, there were 1,783, 2,840, 2,419, and 1,880 upregulated DEGs and 2,179, 2,561, 3,089, and 2,834 downregulated DEGs in each library pair, respectively (Fig. 5b).

In order to investigate the metabolic pathways and aroma related genes, we selected the comparison of HF4-vs-LF4 for further research, in which the upregulation DEGs were much more than downregulation DEGs. Kyoto Encyclopedia of Genes and Genomes (KEGG) pathway analysis was used to identify the critical metabolic pathways of aroma components synthesis in ‘HFBZ’ fruit. The KEGG enrichment top 20 was listed in Fig. 5c, with significant enriched pathways putatively identified as plant hormone signal transduction. Aroma component synthesis pathways included linoleic acid and linolenic acid metabolism were also significantly enriched in KEGG pathway; however, amino acid metabolism was not detected in the KEGG enrichment top 20. Generally, the KEGG enrichment analyses indicated that fatty acids metabolism, rather than amino acid metabolism, contributed most to the aroma compound biosynthesis in ‘HFBZ’ fruit.

The LOX pathway is one of main catabolism of fatty acids for ester biosynthesis. To clarify potential DEGs which may participate in volatile aroma biosynthesis, we further screened the DEGs involved in LOX pathway from the total 5,401 DEGs in HF4-vs-LF4. There were 12 candidate genes that belong to four gene families: *LOXs* (*LOX1a*, *LOX2a*, *LOX2b*, *LOX2c*, *LOX3*, *LOX5a*, and *LOX7a*), two *HPL* (*HPL1* and *HPL2*), two *ADH* (*ADH1* and *ADH2*), and one *AAT* (*AAT1*) genes (Fig. 5d and Supplementary Table S3). Notably, compared with ‘LFBZ’ fruit, *LOX1a*, *LOX2a*, *LOX5a*, *LOX7a*, *ADH1*, and *ADH2* exhibited higher expression level while *LOX2b*, *LOX2c*, *LOX3*, *HPL1*, *HPL2*, and *AAT1* had lower expression in ‘HFBZ’ fruit. Among the upregulation DEGs, in ‘HFBZ’ fruit, *LOX2a*, *LOX5a*, and *ADH1* initially increased and then decreased but *LOX1a* was on the contrary. The gene expression of *LOX7a* increased continually while *ADH2* declined gradually. The above results suggested that LOX pathway was involved in the formation of aroma components in ‘HFBZ’ fruit.

### Expression level of volatile biosynthesis related genes after harvest

**Fig. 6:**
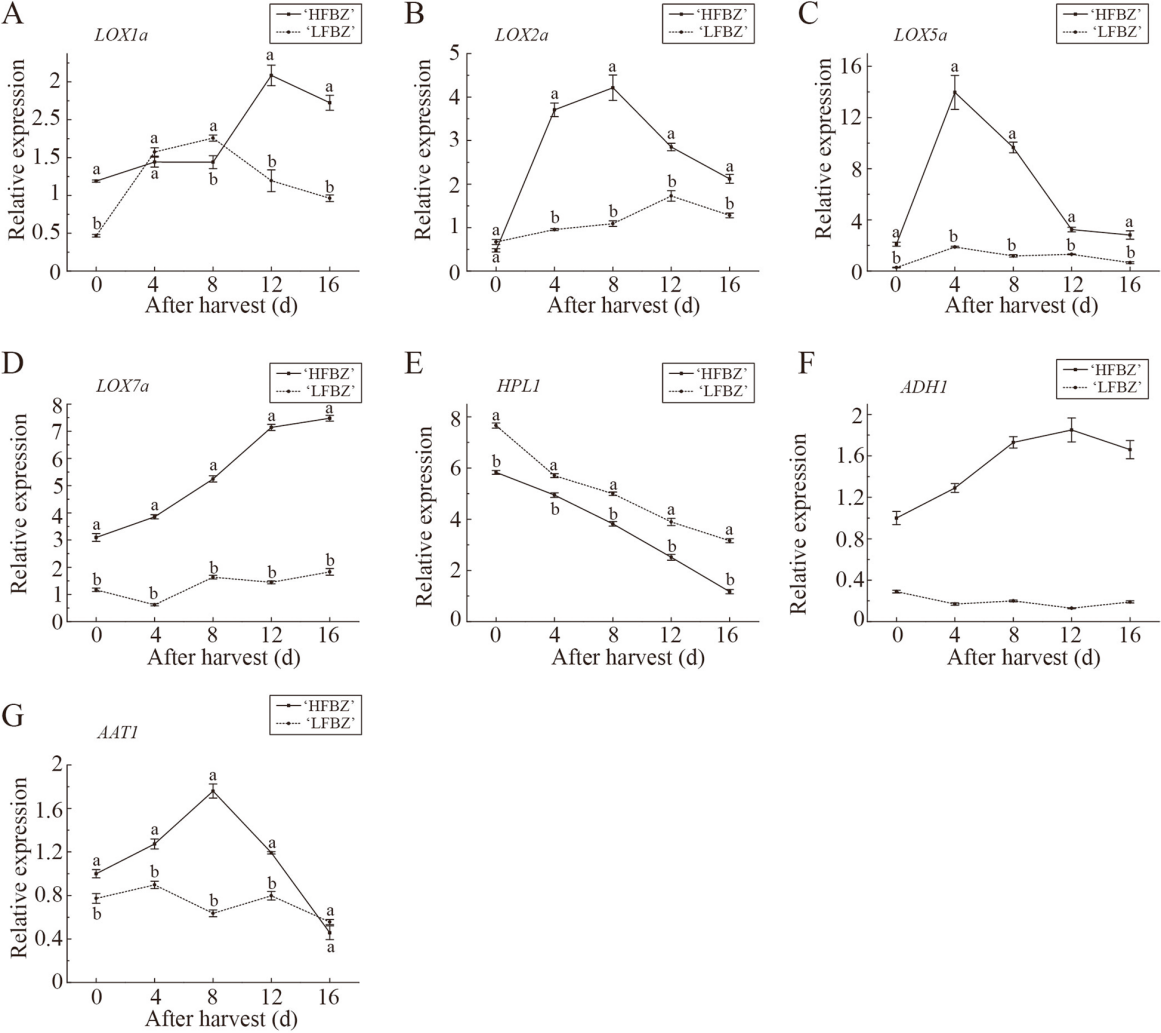
RT-qPCR validated candidate aroma related genes in ‘HFBZ’ and ‘LFBZ’ fruit after harvest. Values represent means of three replicates; bars represent standard deviation of the three replicates. Lowercase letters, a and b, represent significant differences according to the independent sample t-test (*p* < 0.05) for each sampling time point

To further screen out potential aroma related genes, we investigated the expression of seven candidate genes related aroma, including five upregulation genes (*LOX1a*, *LOX2a*, *LOX5a*, *LOX7a*, *ADH1*) and two other member genes (*HPL1* and *AAT1*) involved in LOX pathway by RT-qPCR. Among the LOX gene family members, the expression levels of *LOX1a*, *LOX2a*, *LOX5a*, and *LOX7a* were higher in ‘HFBZ’ fruit than in ‘LFBZ’ fruit (Fig. 6). Different from the RNA-seq result, no significant difference of the *LOX1a* expression level was observed between ‘HFBZ’ and ‘LFBZ’ fruit (Fig. 6a). Interestingly, the *LOX1a* of ‘LFBZ’ fruit rapidly increased before day 4 while slightly changed in ‘HFBZ’ fruit before day 8, suggesting the *LOX1a* might had little influence on the aroma synthesis of ‘HFBZ’ fruit after harvest. The expression profiles of *LOX2a*, *LOX5a*, *LOX7a*, *HPL1*, and *ADH1* genes in ‘HFBZ’ fruit were corresponding to the RNA-Seq results. Among the five genes, the expression levels of *LOX2a* and *LOX5a* increased rapidly as the fruit post-ripening, in which the aroma components accumulated rapidly as well (Fig. 6b and c). Furthermore, the *LOX2a* shared an identity of 71.27% with the grapevine *VvLOXO* gene (FJ858257), which has been categorized as type-2 13-LOX; the *LOX5a* exhibited high similarity towards the hazelnut *CaLOX1* gene (AJ417975, 79.50%), which encoded a functional type-1 9-LOX. Regarding the *ADH1* gene, it increased progressively and reached a peak at day 12 in ‘HFBZ’ fruit, and its expression level was much high than that in ‘LFBZ’ fruit (Fig. 6f). Different from the RNA-seq results, *AAT1* gene expression was higher in ‘HFBZ’ fruit (Fig. 6g), and there was a positive correlation between *AAT1* expression and total volatiles (Supplementary Table S4). Overall, the above results verified that the *LOX2a*, *LOX5a*, *ADH1*, and *AAT1* genes involving in the LOX pathway may contribute most to aroma biosynthesis in ‘HFBZ’ fruit.

### Determination of the major enzyme activities related to the LOX pathway in Binzi fruit after harvest

**Fig. 7:**
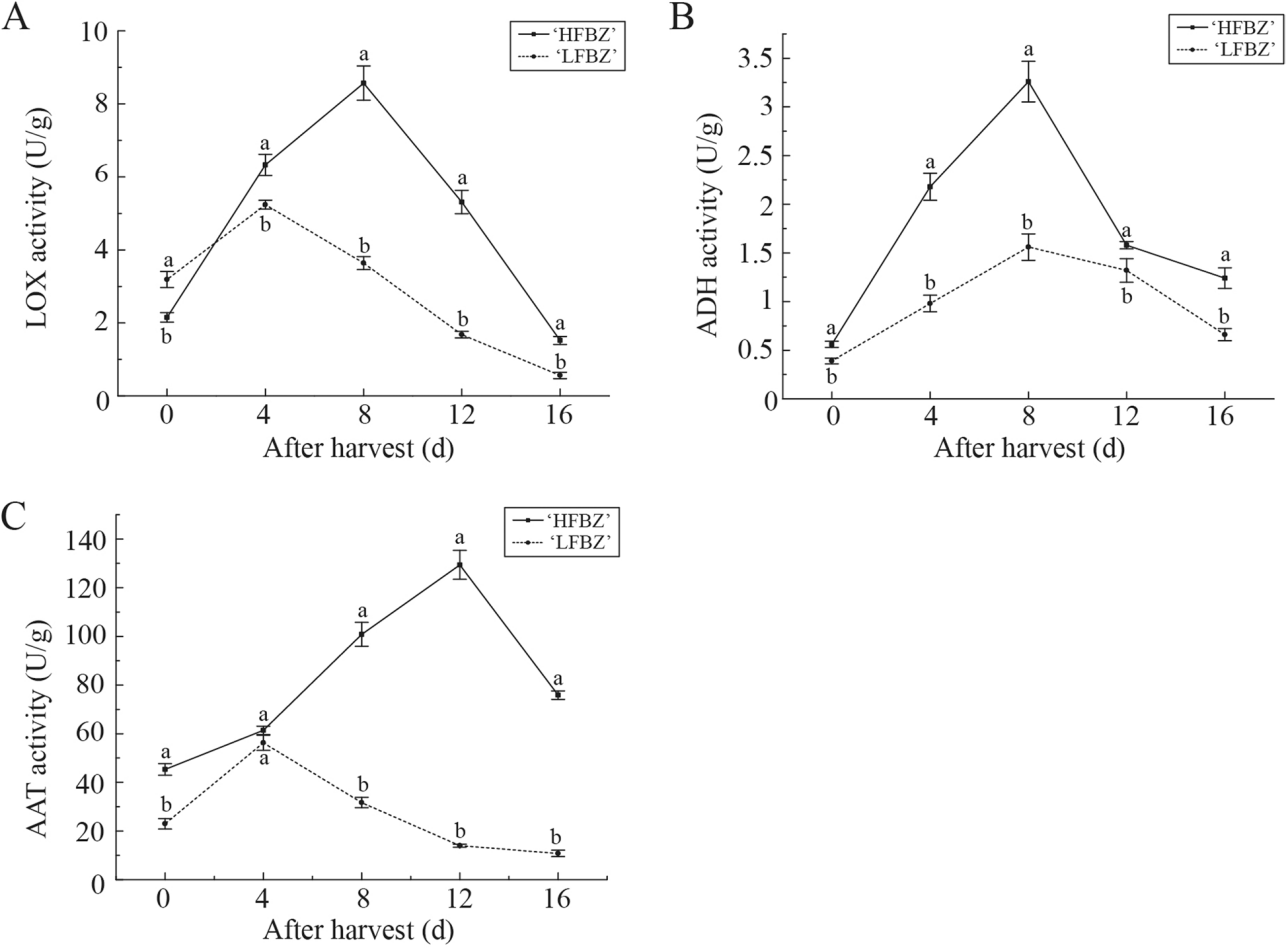
Variation in enzyme activities in ‘HFBZ’ and ‘LFBZ’ fruit after harvest. **a** LOX activity in ‘HFBZ’ and ‘LFBZ’ fruit after harvest. **b** ADH activity in ‘HFBZ’ and ‘LFBZ’ fruit after harvest. **c** AAT activity in ‘HFBZ’ and ‘LFBZ’ fruit after harvest. Values represent means of three replicates; bars represent standard deviation of the three replicates. Lowercase letters a and b represent significant differences according to the independent sample t-test (*p* < 0.05) for each sampling time point

Given that the genes involving in the LOX pathway contributed to aroma biosynthesis, the enzyme activities of LOX, ADH, and AAT were detected. As shown in Fig. 7, the activities of the three enzymes increased progressively at first and then decreased in ‘HFBZ’ and ‘LFBZ’ fruit; however, their activities were higher in ‘HFBZ’ fruit than those in ‘LFBZ’ fruit. In ‘HFBZ’ fruit, LOX and ADH activities rapidly increased and reached a peak at day 8, in consistent with the esters most abundant; while AAT activity gradually increased and reached a peak at day 12. Furthermore, there was a positive correlation between the LOX activity with the total volatiles and esters in ‘HFBZ’ fruit (Supplementary Table S4). Thus, it can be inferred that these enzyme activities involved in LOX pathway was responsible for the aroma quality of ‘HFBZ’ fruit after harvest.

## Discussion

Post-harvest ripening plays a vital role in releasing volatile aroma components, especially in respiratory climacteric fruit, through a series of biochemical reactions [25]. Binzi fruit, known for its attractive aroma, is typical respiratory climacteric fruit with a post-harvest ripening (Fig. 1e and f). The dynamic change in the volatile aroma of Binzi fruit after harvest has not been reported. In this study, we investigated the changes in the components and contents of volatiles, linoleic acid and linolenic acid, amino acids, critical genes related to aroma, and key enzyme activities after harvest.

### Dynamic change in the volatile aroma of Binzi fruit after harvest

Fruit aroma is affected by many factors, among which species and cultivar are especially important. Many studies have explored the differences in aroma compounds in apple, strawberry, cucumber, and other fruit [8, 10, 26, 27]. In this study, a total of 44 and 39 volatile components were identified in ‘HFBZ’ and ‘LFBZ’ fruit at harvest, respectively (Fig. 2a and b). Although the two Binzi cultivars had similar volatile compositions, total volatile components were higher in ‘HFBZ’ than in ‘LFBZ’ fruit (Fig. 2e). Therefore, high accumulation of volatile compounds may contribute to the aroma formation in ‘HFBZ’ fruit. Ethylene plays a vital role in promoting numerous metabolic processes, many of which may contribute to the synthesis of substrates used in the formation of esters [13, 28]. The maximum ester contents of two cultivars occurred near the peaks of ethylene production, which was corresponding to other reports [11, 29]. Previous study found that fruit respiration provided the necessary energy for the synthesis of aroma precursors [30]. The total volatiles increased rapidly at day 4, when the respiration rate reached its peak (Figs. 1e and 2e). Thus, ‘HFBZ’ is a typical climacteric fruit, and post-harvest ripening contributes to the formation of the typical aroma components.

Considerable efforts have been made in determining the aroma components in different climacteric fruits, such as pear, papaya, cherry tomato, and apple [21, 22, 24, 31]. In present study, ethyl acetate, ethyl butanoate, hexyl 2-methylbutyrate, (*E*)-2-hexenal, and 1-hexanol were major aroma components in ‘HFBZ’ fruit juice (Supplementary Table S1). Ethyl acetate and ethyl butanoate was recognized as important contributor to the aroma synthesis in peach fruit [32]. Given that the hexyl 2-methylbutyrate was responsible for the strong ‘fruity’ note of ‘Honeycrisp’ apple [9], we speculated that the ‘fruity’ note of ‘HFBZ’ fruit was also attributed to the hexyl 2-methylbutyrate. The C6 volatiles, (*E*)-2-hexenal and 1-hexanol, were also most prominent in ‘Korla fragrant’ pears and ‘Honeycrisp’ apple [9, 24]. Interestingly, tissues disruption contributes to the formation of aldehydes from free fatty acids by combining lipases, lipids, and the enzymes of LOX pathway [13]. In ‘Jonagold’ apple fruit, (*E*)-2-hexenal from disrupted tissue increased during ripening [25]. Thus, the content of (*E*)-2-hexenal in juice is different from the intact fruit. Taken together, it is suggested that hexyl 2-methylbutyrate was the most potent aroma component, followed by ethyl acetate, ethyl butanoate, (*E*)-2-hexenal, and 1-hexanol in ‘HFBZ’ fruit.

### Fatty acids and amino acids act as precursors to the aroma formation of Binzi fruit

Fatty acids are essential components of membranes and serve as precursors of important aroma substances [33]. Most fatty acids were divided into polar lipid, neutral lipid, and free fatty acid fraction [25]. Furthermore, the free fatty acid fraction was low in immature fruit but rapidly increased approximately fourfold as fruit ripening [29]. Previous study found that the reduction of aroma esters was positive correlated with the variation of oleic acid, linoleic acid, and linolenic acid contents in pears [22]. Oxidation of linoleic acid by LOX enzymes yields hexanal and linolenic acid yields (*Z*)-3-hexenal and (*E*)-2-hexenal [25]. In the present study, linoleic acid and linolenic acid increased greatly with post-ripening and then declined evidently, which corresponded to the variation of ester content in ‘HFBZ’ fruit (Fig. 4a and b). Consistently, our RNA-seq results indicated that the linoleic acid and linolenic acid metabolism were significantly enriched in KEGG pathway from the comparison of ‘HFBZ’ and ‘LFBZ’ fruit (Fig. 5c). It is inferred that the fatty acid metabolism may contribute to the formation of volatile esters in ‘HFBZ’ fruit.

We also found that esters were the main aroma components produced in Binzi fruit (Fig. 2). The esters are largely composed of either straight-chain (SC) or branched-chain (BC) alkyl (alcohol-derived) and alkanoate (acid-derived) groups [34]. Branched-chain ester precursors have been proposed to be derived from branched-chain amino acids (BCAAs), including Leucine (Leu), Ile, and Val [35, 36]. During apple fruit ripening, Ile has been reported to accumulate, but not the other BCAAs [20]. Sugimoto et al. reported that asparagine (Asn), Asp, glutamate (Glu), and Ser were the major amino acids in ripening ‘Jonagold’ fruit [18]. However, in ‘HFBZ’ fruit, Asp had the highest proportion followed by Val, Ala, Ser, and Ile (Fig. 4d). A previous study found that the addition of labeled Leu, Ile, and Val produced different ester patterns in different apple cultivars [37]. However, no significant difference was found in Val and Ile contents between the ‘HFBZ’ and ‘LFBZ’ fruit. Thus, it seemed that the effect of branched-chain amino acids on the aroma difference between ‘HFBZ’ and ‘LFBZ’ fruit was not significant.

### Expression changes of aroma related genes in Binzi fruit after harvest

In apple, LOX pathway is one of the main enzymatic systems in the catabolism of fatty acids for the formation of C6 and C9 aldehydes, alcohols, and esters [15]. The LOX pathway consists of the sequential action of lipase, LOX, HPL, ADH, and AAT [7]. In this study, 12 candidate genes that belonged to four gene families were screened out (Fig. 5d). In pear fruit, *LOX1* and *PpLOX3* are closely related to the synthesis of esters [38]. Overexpression of the tomato *ADH2* gene contributed to improve fruit flavor by increasing the contents of alcohols, particularly 3Z-hexenol [39]. In apple, MdAAT1 functions in key esters synthesis [40]. In this study, RT-qPCR results showed that the *AAT1* gene expression was higher level in ‘HFBZ’ fruit, and correlation analysis certificated the positive correlation between the *AAT1* gene expression and total volatiles (Fig. 6 g and Supplementary Table S4). Based on the above results, we speculated the *LOX2a*, *LOX5a*, *ADH1*, and *AAT1* genes involved in the LOX pathway may contribute to aroma biosynthesis in ‘HFBZ’ fruit.

LOX enzyme catalyzes the oxygenation of polyunsaturated fatty acids to generate hydroperoxide and are classified into 9-LOX or 13-LOX [7, 41, 42]. The 13-LOX enzyme activity contributed to the apple aroma due to the formation of precursors of C6 volatile compounds [43]. Meanwhile, 9-LOX enzyme yielded C9 aldehydes, which were important aroma compounds in cucumber (*Cucumis* ⋅ *sativ*us) [44]. In ‘HFBZ’ fruit, *LOX2a* shared an identity of 71.2% with the grapevine *VvLOXO* gene (FJ858257), which has been categorized as type-2 13-LOX [45]; *LOX5a* exhibited high similarity towards the hazelnut *CaLOX1* gene (AJ417975, 79.50%), which encoded a functional type-1 9-LOX [46]. The (*E*)-2-hexenal in ‘HFBZ’ fruit may be largely generated from the action of LOX2a on fatty acid. Schiller et al. found *MdLOX1*, responsible for majority of LOX activity, contributes to aroma formation in apple fruit [47]. However, it is the *LOX2a*, rather than *LOX1a*, corresponding to the variation trend of LOX activity in ‘HFBZ’ fruit after harvest (Supplementary Table S4). Additionally, the activities of LOX and ADH in ‘HFBZ’ fruit reached their peak at day 8 (Fig. 7a and b), which corresponded to the variation of esters. Even though AAT activity reached a peak 4 d later (Fig. 7c), it also played an important role in volatile compound synthesis by catalyzing alcohol esterification reactions. In conclusion, LOX, ADH, and AAT enzymes involved in LOX pathway are vital for aroma biosynthesis in ‘HFBZ’ fruit.

## Conclusion

The aroma compounds of Binzi fruit were comprehensively analyzed for the first time in the present study. Two Binzi cultivars (‘HFBZ’ and ‘LFBZ’) were selected to explore the aroma metabolism using physiological, biochemical, and molecular methods. In ‘HFBZ’ and ‘LFBZ’ fruit, peaks of respiration rate and ethylene production occur after harvest, and a total of 44 and 39 volatile components were detected at harvest day, respectively. Compared with the ‘LFBZ’ fruit, the ‘HFBZ’ fruit produces full aroma, accompanied by higher contents of aroma compounds, fatty acids (linoleic acid and linolenic acid), and branched-chain amino acids (isoleucine and valine), with higher activities of LOX, ADH, and AAT, and higher expression of *LOX2a*, *LOX5a*, *ADH1*, and *AAT1*. Besides, esters are the main aroma components of ‘HFBZ’ fruit, and hexyl 2-methylbutyrate was the most potent aroma component, followed by ethyl acetate, ethyl butanoate, (*E*)-2-hexenal, and 1-hexanol in ‘HFBZ’ fruit. Moreover, RNA-seq result showed that linoleic acid and linolenic acid metabolism were significantly enriched in KEGG pathway. Thus, it can be deduced: (1) it is the content of aroma components, rather than the variety, that is vital to the different odor in ‘HFBZ’ and ‘LFBZ’ fruit; (2) the ‘fruity’ note of ‘HFBZ’ fruit is attributed to the hexyl 2-methylbutyrate; (3) compared with amino acid metabolism, fatty acid metabolism is more important for aroma synthesis of ‘HFBZ’ fruit; (4) *LOX2a*, *LOX5a*, *ADH1*, and *AAT1* genes involved in the LOX pathway may contribute to aroma biosynthesis in ‘HFBZ’ fruit. Overall, the research firstly investigated the aroma components, and screened four candidate genes related aroma biosynthesis of Binzi fruit. Our findings will contribute to the understanding of the mechanism of aroma formation and provided an insight into the fragrant nature of *Malus* species.

## Methods

### Plant materials and treatments

‘HFBZ’ and ‘LFBZ’ fruit were chosen due to their vast differences in flavour profile and similar genetic background. Binzi fruit of uniform size, without mechanical damage and disease were harvested from one orchard in mountainous area of Beijing, at the commercial maturity time when the SSC was about 14%. To monitor post-harvest changes, the sampled fruit were stored at an ambient temperature (20 ± 1 °C) and relative humidity (RH, 8% – 8%) for 16 d. The fruit physiological indices, enzyme activities, and aroma components were analyzed at 4 d intervals. Meanwhile, the fruit were instantly frozen in liquid nitrogen and stored at − 80 °C for RNA-sequence (RNA-seq) analysis, amino acid, extraction of fatty acid, and determination of saccharide content. All measurements were repeated three times for six fruit in each biological replicate.

### Determination of fruit anthocyanin content, firmness, SSC, and saccharide content

Fruit anthocyanin content was determined according to the method described previously [48]. Approximately 2 g fruit power was mixed with 5 mL cold methanol with 1% HCl and kept overnight in the dark at 4 °C. The mixture was centrifuged at 10,000 *g* for 15 min at 4 °C. Then, the absorbances were measured at 530 nm, 620 nm, and 650 nm using a spectrophotometer (UV752, Heerpu, Shanghai, China). The anthocyanin content was calculated with the following formula:

$$\mathrm{Anthocyanin}\;\mathrm{content}\;\left(\mathrm{mmol}\;\mathrm{kg}^{-1}\right)\;=\;{\mathrm{OD}}_\lambda/\xi_\lambda\times\;\mathrm V/\mathrm m\times10^{9},$$

$$\mathrm{optical}\;\mathrm{density}\;\left({\mathrm{OD}}_\lambda\right)=\left(\mathrm A530\;-\;\mathrm A620\right)\;-\;0.1\;\times\;\left(\mathrm A650\;-\;\mathrm A620\right),\;\xi_{\lambda}\;=\;4.50\;\times\;10^{4}.$$

Firmness was measured after removing the fruit peel by using a fruit hardness analyzer (FHM-5, Takemura Electric Works Ltd., Tokyo, Japan). SSC was determined using a digital refractometer (TD-45, Zhejiang, China).

The contents of sucrose, glucose, and fructose in Binzi fruit were determined using reverse-phase HPLC with a Sugar‐Pak™ I column (6.5 mm × 300 mm ⋅ 10 μm, Waters, Milford, MA, USA), following the previous method [49]. 1 g fruit sample was ground into a powder in liquid nitrogen. And the powder was then mixed with 10 mL 8% ethyl alcohol and kept at 80 °C for 30 min. The mixture was centrifuged at 10,000 *g* for 10 min. The supernatant was transferred into a new tube and evaporated. The residue was dissolved in purified water and filtered through Supelclean LC-18 SPE (Sigma Aldrich, USA). The standard calibration curves of the saccharide content were prepared by gradient dilution for five times, with concentrations of 0.5, 0.25, 0.1, 0.05, and 0.025 g/L for D(+)-glucose, D(−)-fructose, and sucrose, respectively. The detection value was measured by refractive index detector (RID). The contents of sucrose, glucose, and fructose were separately evaluated based on the ratio of the standard calibration.

### Determination of ethylene production and respiratory rate

Nine fruit with uniform size and ripeness were divided randomly into three groups. And three fruit were weighed and placed in a 1 L sealed container for 2 h at 20 ± 1 °C. The 0.5 mL headspace was subsequently sampled and evaluated using a gas chromatograph (GC; Agilent-7890, Agilent Technologies, USA) equipped with a flame ionization detector. Nitrogen was used as carrier gas. Ethylene production was expressed as ng/kg·s. The respiratory rate (µg/kg·s) was determined using a gas analyzer (PBI Dansensor Checkmate II, Denmark).

### Determination of volatile components

Six fruit were used to extract the volatile components using solid-phase microextraction. After adding 1 g D-gluconolactone and 3 g polyvinylpolypyrrolidone (PVPP), 20 g fruit were squeezed to obtain the juice by a homogenizer, then incubated at 4 °C for 2 h. After filtering through six layers of gauze, 5 mL of fruit juice was transferred to a 20 mL vial before adding 1 g of NaCl. Before the vial was sealed, 10 µL of 4-methyl-2-pentanol (1.068 g/L) was added as an internal standard. The solid-phase microextraction (SPME) conditions were as follows: equilibration at 40 °C for 30 min, followed by desorption at 250 °C for 8 min.

Gas Chromatography-Mass Spectrometer (GC–MS; 7890 A–5975 C, Agilent, Santa Clara, CA, USA) with an HP-INNOWAX (60 m × 0.25 mm × 0.25 μm, J & W scientific, USA) was used to analyze the volatile components. The conditions for GC were as follows: injector, 250 °C; initial oven temperature was 50 ℃, held for 1 min and then increased to 220 °C at a rate of by 3 °C/min. Helium was used as carrier gas at a rate of 1 × 10^− 3^ L/min. The MS conditions were as follows: ion source temperature, 230 °C; electron energy, 70 eV; GC–MS interface zone, 280 °C; scan range, m/z 30–350. The NIST/Wiley MS Search 2.0 mass spectral library was used to identify volatile compounds. The volatile content was evaluated based on the ratio of the volatile peak area to the peak area of 4-methyl-2-pentanol.

### Measurement of linoleic acid and linolenic acid

Fatty acids were extracted by GC–MS [50]. Approximately 3 g of fruit samples were ground into a powder in liquid nitrogen, added to 5 mL extraction solution (chloroform / methanol, 2:1, v/v), and then subjected to ultrasonic extraction for 20 min. The mixture was centrifuged at 10,000 *g* for 20 min at 20 °C. The supernatant was transferred into a new glass tube, after which 5 mL of chloroform and NaCl (0.76%, w/v) was added, and the organic phase was dried by a nitrogen stream. The residue was dissolved with 1 mL hexane and methylated with 0.4 M KOH-methanol solution. A GC–MS (Agilent 7890 A/5975 C, Agilent Technologies, USA) instrument equipped with a HP-5ms column (0.25 mm I.D ⋅ 30 m ⋅ 0.25 μm). Helium was used as the carrier gas. The injection volume was 1 µL, and the split ratio was 50:1. The conditions for gas chromatography were as follows: injector, 270 °C; initial temperature 130 °C, increased by 15 °C/min to 280 °C and maintained for 5 min. The MS conditions were as follows: ion source, 230 °C; electron energy, 70 eV; GC–MS interface zone, 250 °C; scan range, m/z 12–550. The linoleic acid and linolenic acid were identified by using the NIST/Wiley MS Search 2.0 mass spectral library.

### Measurement of amino acid content

For the amino acid content, 5 g of fruit sample was ground into a powder in liquid nitrogen. The powder was then mixed with 10 mL 0.02 mol/L HCl (cold) and kept at 4 °C for 12 h. After centrifugation at 12,000 *g* for 15 min at 4 °C, the supernatant was mixed with 4% sulfosalicylic acid with a ratio of 1:1. The mixture was filtered through a 0.22 μm membrane (Millipore, Billerica, MA, USA), and then subjected to an automatic amino acid analyzer (L-8900, Hitachi, Japan). Separation was performed using an ion exchange column. The mobile phase was citrate buffer at a flow rate of 4 × 10^− 4^ L/min. The ultraviolet-visible light detector wavelength was set at 440 and 570 nm.

### RNA extraction and RNA-seq

Six fruits from each treatment (day 0, day 4, day 8, and day 12 after harvest) were prepared for transcriptome sequencing. Total RNA was extracted using a RNeasy Plant Mini Kit (Qiagen, Dusseldorf, Germany). RNA purity and quantification were evaluated using a NanoDrop 2000 spectrophotometer (Thermo Scientific, USA). RNA integrity was assessed using an Agilent 2100 Bioanalyzer (Agilent Technologies, Santa Clara, CA, USA). The libraries were constructed using TruSeq Stranded mRNA LT Sample Prep Kit (Illumina, San Diego, CA, USA) and sequenced on an Illumina NovaSeq 6000 platform by OE Biotech Co., Ltd. (Shanghai, China). Hierarchical cluster analysis of differentially expressed genes (DEGs) was performed to investigate the genes expression pattern of different samples. After reads ressembled by StringTie software, novel transcripts identification was performed by comparing the *Malus* ⋅ *domestica* genome (ftp://ftp.ncbi.nlm.nih.gov/genomes/all/GCF/002/114/115/GCF_002_114_115.1_AS_M211411v1/GCF_002114115.1_ASM211411v1_genomic.fna.gz) using Cuffcompare software. ASprofile software was used to analyse the alternatively splicing of differentially regulated transcripts isoforms. According to the expression level of the protein-coding gene among the different treatments, a significant difference in expression was obtained as |log2 fold change| ≥ 2. The RNA-seq was performed with three repeats.

### Relative gene expression assay

RT-qPCR was performed using the Roche LightCycler® 96 Real-Time PCR System (Roche Diagnostics GmbH, Mannheim, Germany). Gene-specific primers were designed using Primer 5 and synthesized using Genewiz (Suzhou, China). Subsequently, the RNA was reverse transcribed using the TransScript First-Strand cDNA Synthesis SuperMix (TransGen Biotech, Beijing, China). PCR conditions were as follows: an initial denaturation at 95 °C for 30 s, followed by 45 cycles of denaturation at 95 °C for 5 s, annealing at 60 °C for 15 s, extension at 72 °C for 10 s, and a final extension at 72 °C for 3 min. The relative expression of the target genes was calculated using the 2^−ΔΔCt^ method. All reactions were conducted in triplicates. The primer sequences of the target and reference genes are listed in Table S5.

### Enzyme activity analysis

LOX activity was assayed according to previous method [50]. 4 g of tissue was ground into a powder in liquid nitrogen, added to 12 mL of cold phosphate buffered saline (PBS) buffer (0.1 M, pH 7.0) and then kept at 4 °C for 1 h. The mixture was centrifuged at 15,000 *g* for 15 min at 4 °C. The supernatant was collected and then put on ice for used to measure LOX activity. The reaction assay system comprised 2.8 mL of 0.05 M PBS buffer (pH 7.0), 0.1 mL of 0.01 M linoleic acid sodium salt solution, and 0.1 mL crude enzyme. The reaction mixture without enzyme solution was used as a control. One unit of LOX activity was defined as a change of 0.01 in absorbance per minute at 234 nm.

ADH activity was measured as previously described [51]. 3 g of tissue powder was homogenized in 5 mL of extracting solution, and then centrifuged at 15,000 *g* for 15 min at 4 °C. The supernatant was put on ice for used to measure ADH activity. The reaction assay system comprised 2.4 mL of 100 mM 2-(4-morpholino) ethanesulfonic acid (MES)–Tris buffer, 0.15 mL of 1.6 mM NADH solution, 0.15 mL of 80 mM acetaldehyde, and 0.3 mL of ADH crude enzyme. One unit of ADH activity was defined as a change of 0.01 per minute at 340 nm.

AAT activity was measured by the method described previously [51]. Three grams of tissue powder was homogenized in 6 mL of extracting solution comprising 85 mM MES buffer (pH 7.0), 5 mM dithiothreitol (DTT), and 1% (w/v) PVPP. The homogenate was centrifuged at 15,000 *g* for 15 min at 4 °C. The supernatant was put on ice for used to measure AAT activity. The reaction assay system comprised 2.5 mL of 5 mM MgCl_2_ solution, 150 µL of 5 mM acetyl-CoA, 50 µL butanol, and 300 µL crude enzyme. The mixture was kept at 35 °C for 15 min, after which 10 mL of 10 mM DTNB was added, and then incubation for 10 min at room temperature. The enzyme activity of AAT was determined by change at 412 nm. One unit of AAT activity was defined as a change of 0.01 per minute at 412 nm.

### Statistical analysis

Statistical analyses were performed using SPSS Statistics v.20, including significant difference analysis and correlation analysis (SPSS Inc., Chicago, IL, USA). Graphs were prepared using Origin 8.1 (MicroCal Software Inc., Northampton, MA) and Adobe Illustrator (CC 2017; Adobe Inc., San Jose, USA). Experimental results are presented as the mean of three biological replicates ± standard deviation.

## Supplementary Information

**Additional file 1:** **Supplementary Fig. 1** Morphology of ‘HFBZ’ and ‘LFBZ’ fruit at harvest. **a** ‘HFBZ’ fruit (Left) and ‘LFBZ’ (Right) fruit. **b** Transverse and longitudinal diameter, and weight of ‘HFBZ’ and ‘LFBZ’ fruit. **c** Anthocyanin content of ‘HFBZ’ and ‘LFBZ’ fruit. The scale bar is 1 cm in a. **Supplementary Fig. 2** Liquid chromatography profiles of sucrose, glucose, and fructose standards. **Supplementary Fig. 3** Chromatograms of free amino acid standard (**a**) and free amino acid content of ‘HFBZ’ (**b**) and ‘LFBZ’ (**c**) fruit at harvest. **Supplementary Table 1.** Compositions and contents of total volatile compounds in ‘HFBZ’ and ‘LFBZ’ fruit after harvest. The total volatile compounds were divided into esters, aldehydes, alcohols, ketones, and others five groups. “0, 4, 8, 12 and 16” respectively represents at day 0, 4, 8, 12 and 16 after harvest. **Supplementary Table 2.** Quality analysis of each sample about RNA-seq. **Supplementary Table 3.** Analyzed expression level of volatile related genes of the FPKM values in ‘HFBZ’ and ‘LFBZ’ fruit after harvest. **Supplementary Table 4.** Correlation analysis of volatile components, crucial aroma related genes, and enzyme activities. **Supplementary Table 5.** Gene-specific primers used for RT-qPCR analysis.

## Data Availability

The datasets generated and/or analysed during the current study are available in the NCBI database repository. The SRA accession number is SUB11241044, and the BioSample accession numbers are SAMN27015323– SAMN27015328. The web link is https://submit.ncbi.nlm.nih.gov/subs/sra/SUB11241044/overview. The data will be shared on reasonable request of the corresponding author.

## Abbreviations

HFBZ: High-fragrant Binzi
LFBZ: Low-fragrant Binzi
GC–MS: Gas Chromatography–Mass Spectrometer
LOX: Lipoxygenase
HPL: Hydroperoxide lyase
ADH: Alcohol dehydrogenase
AAT: Alcohol acyltransferase
PRC: Peaks of respiratory climacteric
DEGs: Differentially expressed genes
KEGG: Kyoto encyclopedia of genes and genomes
BCAAs: Branched-chain amino acids
Leu: Leucine
Ile: Isoleucine
Val: Valine
Asn: Asparagine
Asp: Aspartic acid
Glu: Glutamate
SSC: Soluble solid content

## Declarations

b) ‘HFBZ’ and ‘LFBZ’ fruits were harvested from one orchard in mountainous area of Beijing, China. And we have permission to collect the fruits.
