# Supplementary material for: Characterization of key aroma compounds and regulation mechanism of aroma formation in local Binzi (Malus pumila × Malus asiatica) fruit
Source: BMC Plant Biol. 2022 Nov 15;22:532. doi: 10.1186/s12870-022-03896-z (PMC9664629; doi:10.1186/s12870-022-03896-z)
Supplement: Supplementary file 1 — Additional file 1: Supplementary Fig. 1 Morphology of ‘HFBZ’ and ‘LFBZ’ fruit at harvest. a ‘HFBZ’ fruit (Left) and ‘LFBZ’ (Right) fruit. b Transverse and longitudinal diameter, and weight of ‘HFBZ’ and ‘LFBZ’ fruit. c Anthocyanin content of ‘HFBZ’ and ‘LFBZ’ fruit. The scale bar is 1 cm in a. Supplementary Fig. 2 Liquid chromatography profiles of sucrose, glucose, and fructose standards. Supplementary Fig. 3 Chromatograms of free amino acid standard (a) and free amino acid content of ‘HFBZ’ (b) and ‘LFBZ’ (c) fruit at harvest. Supplementary Table 1. Compositions and contents of total volatile compounds in ‘HFBZ’ and ‘LFBZ’ fruit after harvest. The total volatile compounds were divided into esters, aldehydes, alcohols, ketones, and others five groups. “0, 4, 8, 12 and 16” respectively represents at day 0, 4, 8, 12 and 16 after harvest. Supplementary Table 2. Quality analysis of each sample about RNA-seq. Supplementary Table 3. Analyzed expression level of volatile related genes of the FPKM values in ‘HFBZ’ and ‘LFBZ’ fruit after harvest. Supplementary Table 4. Correlation analysis of volatile components, crucial aroma related genes, and enzyme activities. Supplementary Table 5. Gene-specific primers used for RT-qPCR analysis. [file 12870_2022_3896_MOESM1_ESM.docx]

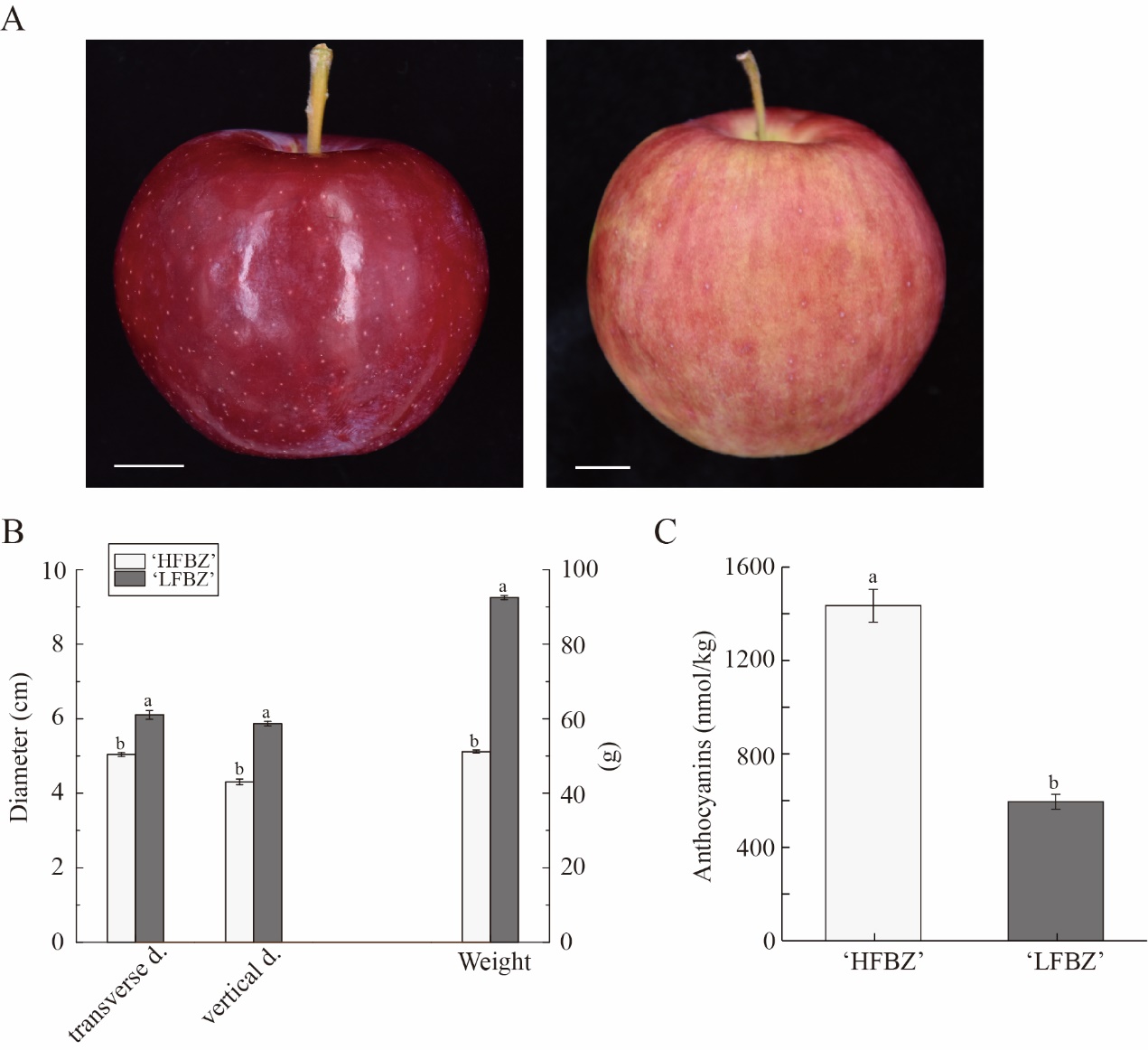
**Supplementary Figure 1.** Morphology of ‘HFBZ’ and ‘LFBZ’ fruit at harvest

**a** ‘HFBZ’ fruit (Left) and ‘LFBZ’ (Right) fruit. **b** Transverse and longitudinal diameter, and weight of ‘HFBZ’ and ‘LFBZ’ fruit. **c** Anthocyanin content of ‘HFBZ’ and ‘LFBZ’ fruit. The scale bar is 1 cm in **a**.


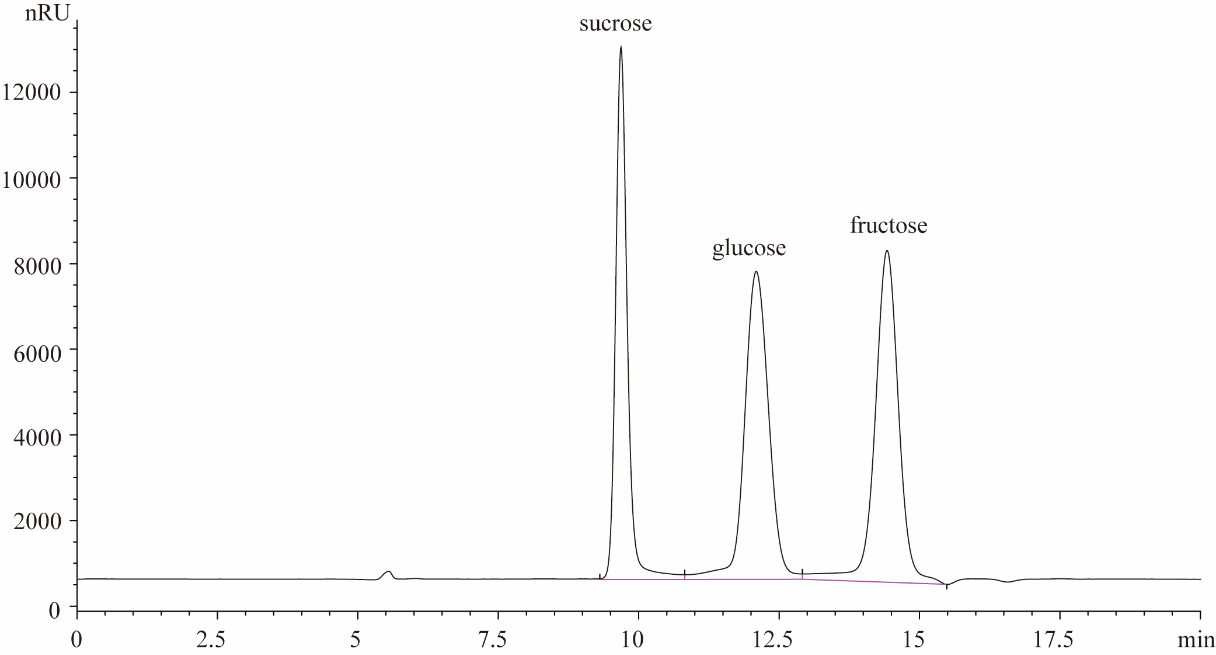


**Supplementary Figure 2.** Liquid chromatography profiles of sucrose, glucose, and fructose standards


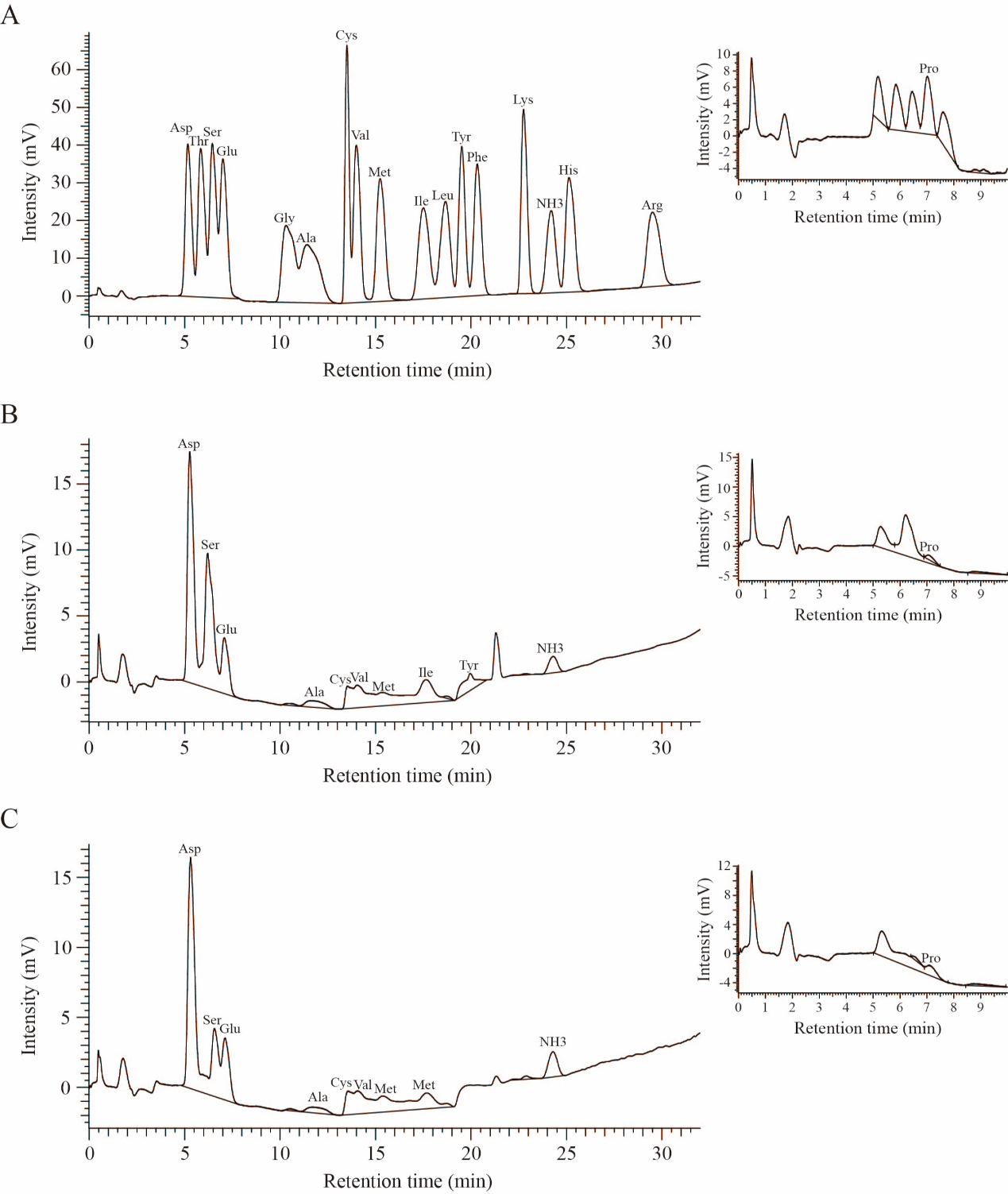
 **Supplementary Figure 3.** Chromatograms of free amino acid standard (**a**) and free amino acid content of ‘HFBZ’ (**b**) and ‘LFBZ’ (**c**) fruit at harvest

**Supplemental Table 1** Compositions and contents of total volatile compounds in ‘HFBZ’ and ‘LFBZ’ fruit after harvest

| Volatile compounds (μg/L) | CAS | ‘HFBZ’ after harvest (d) | | | | | ‘LFBZ’ after harvest (d) | | | | |
| --- | --- | --- | --- | --- | --- | --- | --- | --- | --- | --- | --- |
|  |  | 0 | 4 | 8 | 12 | 16 | 0 | 4 | 8 | 12 | 16 |
| **Esters** | | | | | | | | | | | |
| **Ethyl Acetate** | 141-78-6 | 1114.75±  89.58 | 4315.70±  309.11 | 6084.14±513.72 | 7389.13±  371.98 | 2377.00±  179.29 | 442.73±  30.48 | 638.77±  40.44 | 438.95±  32.71 | 315.49±  24.16 | 327.88±  25.24 |
| **Ethyl butanoate** | 105-54-4 | 593.80±  46.15 | 688.45±  39.71 | 475.67±  32.55 | 340.89±  20.56 | 172.19±  10.39 | 8.49±  0.57 | 35.58±  2.59 | 24.36±  1.69 | 14.37±  0.12 | 16.71±  1.13 |
| Ethyl 2-methylbutanoate | 7452-79-1 | 158.87±  9.59 | 144.01±  10.37 | 112.35±  9.73 | 131.39±  10.18 | 57.18±  5.61 | 5.76±  0.33 | 101.98±  6.88 | 43.80±  3.51 | 39.25±  2.29 | 48.21±  3.17 |
| **Hexyl 2-methylbutyrate** | 10032-15-2 | 2160.73±  220.31 | 2486.85±  197.53 | 2173.30±  193.69 | 1611.69±  112.54 | 1085.35±  69.88 | 1612.50±  113.99 | 1630.53±  129.33 | 1405.14±  112.77 | 1249.53±  68.28 | 1374.42±  93.75 |
| Methyl hexanoate | 106-70-7 | 7.17±  0.28 | 20.90±  1.18 | 35.72±  2.16 | 26.00±  1.63 | 16.57±  1.27 | 1.91±  0.15 | 1.54±  0.12 | 1.80±  0.13 | 1.37±  0.12 | 1.31±  0.10 |
| 2-Methylbutyl ethanoate | 624-41-9 | ND | 4.19±  0.28 | 3.49±  0.31 | ND | ND | 6.53±  0.37 | 28.67±  1.98 | 33.82±  2.59 | 2.66±  0.21 | 10.99±  0.67 |
| Ethyl hexanoate | 123-66-0 | 50.20±  1.39 | 67.20±  7.51 | 83.73±  3.29 | 81.10±  4.55 | 33.99±  2.57 | 1.26±  0.08 | 1.97±  0.12 | 2.69±  0.17 | 1.74±  0.12 | 1.85±  0.11 |
| Hexyl acetate | 142-92-7 | 3.68±  0.20 | 2.48±  0.17 | 2.67±  0.10 | 2.09±  0.11 | 1.44±  0.17 | 1.49±  0.13 | 1.41±  0.08 | 1.28±  0.10 | 1.33±  0.08 | 1.34±  0.08 |
| Ethyl 3-hexenoate | 2396-83-0 | 0.80±  0.05 | 1.92±  0.12 | 1.50±  0.06 | 1.22±  0.09 | 1.11±  0.04 | ND | ND | ND | ND | ND |
| Ethyl heptanoate | 106-30-9 | 0.90±  0.09 | 0.98±  0.06 | 0.87±  0.07 | 0.30±  0.02 | 0.73±  0.06 | 0.12±  0.01 | 0.21±  0.02 | 0.19±  0.01 | 0.21±  0.02 | 0.68±  0.04 |
| Ethyl octanoate | 106-32-1 | 5.75±  0.31 | 6.27±  0.59 | 6.01±  0.51 | 6.11±  0.47 | 5.32±  0.33 | 4.81±  0.26 | 5.74±  0.37 | 6.83±  0.44 | 5.26±  0.03 | 8.81±  0.47 |
| Ethyl 3-hydroxybutyrate | 5405-41-4 | 5.06±  0.16 | 17.58±  1.31 | 16.39±  1.59 | 21.42±  1.56 | 22.85±  1.58 | 1.80±  0.15 | 7.76±  0.64 | 11.82±  1.06 | 8.10±  0.59 | 10.94±  0.71 |
| Hexyl hexanoate | 6378-65-0 | 3.02±  0.29 | 1.93±  0.13 | 1.63±  0.14 | 1.51±  0.13 | 1.43±  0.07 | 1.61±  0.11 | 1.57±  0.13 | 1.55±  0.12 | 1.43±  0.11 | 1.49±  0.12 |
| Ethyl decanoate | 110-38-3 | 1.43±  0.09 | 1.52±  0.08 | 1.55±  0.72 | 1.48±  0.09 | 1.51±  0.09 | ND | 1.43±  0.10 | 1.74±  0.13 | 1.50±  0.08 | 1.91±  0.13 |
| Ethyl trans-4-decenoate | 76649-16-6 | 1.49±  0.17 | 1.53±  0.12 | 1.58±  0.10 | 1.52±  0.09 | 1.43±  0.09 | 1.45±  0.13 | 1.46±  0.06 | 1.43±  0.06 | 1.47±  0.12 | 1.43±  0.08 |
| Ethyl succinate | 123-25-1 | 3.59±  0.18 | 3.22±  0.39 | 28.30±  2.01 | 13.42±  1.06 | 18.28±  1.21 | 19.86±  1.25 | 31.81±  2.51 | 5.33±  0.39 | 3.26±  0.27 | 11.61±  1.03 |
| Ethyl 3-hydroxyhexanoate | 2305-25-1 | 1.91±  0.21 | 3.02±  0.24 | 3.11±  0.16 | 2.35±  0.18 | 1.84±  0.21 | 1.13±  0.09 | 1.57±  0.13 | 1.70±  0.13 | 1.21±  0.07 | 1.62±  0.12 |
| Ethyl phenylacetate | 101-97-3 | 1.76±  0.10 | 2.72±  0.19 | 2.10±  0.17 | 2.13±  0.16 | 2.01±  0.14 | ND | ND | ND | ND | ND |
| **Aldehydes** | | | | | | | | | | | |
| **(*E)*-2-hexenal** | 6728-26-3 | 1223.85±  96.88 | 2131.63±  139.53 | 1903.60±  125.26 | 1510.47±  132.09 | 775.01±  53.95 | 1286.10±  95.45 | 2095.42±  166.54 | 1010.22±  67.66 | 884.14±  62.11 | 1178.89±  89.44 |
| Octanal | 124-13-0 | 1.16±  0.17 | 2.87±  0.17 | 1.09±  0.08 | 1.10±  0.06 | 1.86±  0.15 | 0.74±  0.04 | 1.22±  0.10 | 0.82  0.04 | 1.79±  0.11 | 1.91±  0.15 |
| Nonanal | 124-19-6 | 4.43±  0.39 | 9.31±  1.06 | 1.41±  0.08 | 4.40±  0.26 | 3.13±  0.27 | 2.90±  0.22 | 0.43±  0.02 | 5.62  0.46 | 0.40±  0.02 | 9.03±  0.73 |
| 2,4-Hexadienal, (E, E)- | 142-83-6 | 57.61±  7.23 | 46.08±  2.74 | 41.49±  3.75 | 30.26±  2.49 | 23.96±  1.59 | 39.75±  2.97 | 28.24±  2.04 | 24.03  1.29 | 21.62±  1.68 | 26.82±  1.94 |
| Decanal | 112-31-2 | 5.00±  0.37 | 5.29±  0.33 | 5.00±  0.35 | 4.83±  0.31 | 4.76±  0.33 | ND | ND | ND | ND | ND |
| Benzaldehyde | 100-52-7 | 4.18±  0.31 | 3.80±  0.19 | 3.09±  0.13 | 2.21±  0.16 | 3.29±  0.21 | 2.83±  0.16 | 4.23±  0.23 | 3.00±  0.18 | 3.42±  0.27 | 4.52±  0.28 |
| 2-Nonenal, (E)- | 18829-56-6 | 5.10±  0.44 | 4.98±  0.27 | 4.93±  0.33 | 4.99±  0.28 | 5.05±  0.37 | 4.89±  0.34 | 5.02±  0.41 | 4.94±  0.37 | 4.87±  0.31 | 4.91±  0.26 |
| **Alcohols** | | | | | | | | | | | |
| 1-Butanol | 71-36-3 | 213.68±  17.62 | 101.00±  8.13 | 44.84±  3.29 | 23.50±  1.95 | 13.44±  0.15 | ND | 24.57±  9.53 | 30.86±  2.11 | ND | 5.43±  0.45 |
| **1-Hexanol** | 111-27-3 | 1313.65±  98.55 | 1122.72±  93.55 | 908.17±  56.53 | 242.49±  18.53 | 77.11±  5.83 | 142.74±  10.44 | 313.08±  19.57 | 293.91±  16.69 | 213.18±  16.44 | 217.90±  16.36 |
| (Z)-3-Hexen-1-ol | 928-96-1 | 7.31±  0.40 | 15.88±  1.07 | 11.90±  0.98 | 2.57±  0.37 | 2.57±  0.17 | ND | ND | ND | ND | ND |
| 2-Hexen-1-ol, (E)- | 928-95-0 | 87.21±  5.14 | 83.86±  3.51 | 42.28±  3.28 | 40.37±  2.33 | 14.46±  1.22 | 174.69±  12.85 | 48.47±  2.31 | 104.85±  6.32 | 0.24±  0.01 | 31.77±  2.49 |
| 1-Octen-3-ol | 3191-86-4 | 1.12±  0.07 | 1.19±  0.09 | 0.85±  0.05 | 0.82±  0.07 | 0.92±  0.05 | 1.07±  0.07 | 1.11±  0.08 | 0.97±  0.06 | 1.09±  0.06 | 1.19±  0.10 |
| 1-Heptanol | 111-70-6 | 3.62±  0.17 | 2.35±  0.18 | 1.95±  0.14 | 0.47±  0.02 | 0.46±  0.03 | 0.96±  0.0 | 1.85±  0.13 | 1.61±  0.11 | 0.93±  0.05 | 1.26±  0.09 |
| Linalool | 78-70-6 | 4.54±  0.27 | 4.51±  0.32 | 4.49±  0.39 | 4.48±  0.29 | 4.49±  0.29 | 4.34±  0.29 | 4.37±  0.29 | 4.38±  0.26 | 4.31±  0.29 | 4.52±  0.37 |
| 1-Octanol | 111-87-5 | 3.19±  0.13 | 2.1±  0.16 | 2.03±  0.15 | 1.70±  0.12 | 1.82±  0.11 | 1.98±  0.11 | 2.05±  0.17 | 1.87±  0.13 | 1.73±  0.14 | 2.16±  0.15 |
| 1-Nonanol | 143-08-8 | 1.24±  0.08 | 1.26±  0.10 | 1.23±  0.07 | 1.21±  0.10 | 1.33±  0.08 | 1.15±  0.08 | 1.21±  0.06 | 1.11±  0.08 | 1.23±  0.07 | 1.24±  0.08 |
| α-Terpineol | 10482-56-1 | 4.77±  0.53 | 4.62±  0.33 | 4.79±  0.31 | 4.76±  0.41 | 4.81±  0.32 | 4.65±  0.39 | 4.85±  0.32 | 4.78±  0.39 | 4.62±  0.25 | 4.87±  0.35 |
| Benzyl alcohol | 100-51-6 | 44.52±  3.79 | 43.44±  29.53 | 86.32±  3.59 | 66.72±  5.91 | 78.91±  5.77 | 90.48±  4.97 | 109.53±  7.55 | 71.18±  4.83 | 41.83±  2.93 | 78.78±  6.71 |
| Phenethanol | 60-12-8 | 49.59±  2.11 | 56.23±  4.01 | 77.99±  5.31 | 70.2±  5.23 | 74.64±  5.27 | 64.27±  5.93 | 69.18±  5.83 | 52.64±  3.93 | 49.27±  3.37 | 85.18±  4.66 |
| **Ketones** | | | | | | | | | | | |
| 6-Methyl-5-hepten-2-one | 110-93-0 | 7.90±  0.93 | 6.59±  0.37 | 5.41±  0.29 | 4.82±  0.23 | 3.80±  0.21 | 6.77±  0.31 | 9.32±  0.49 | 10.76±  0.97 | 4.56±  0.31 | 11.33±  0.87 |
| Acetophenone | 21070-22-4 | 2.04±  0.37 | 2.01±  0.13 | 1.97±  0.13 | 1.87±  0.17 | 1.98±  0.15 | 1.88±  0.14 | 2.10±  0.11 | 1.99±  0.13 | 1.92±  0.11 | 2.07±  0.22 |
| β-Damascenone | 23726-91-2 | 22.33±  1.95 | 19.26±  1.33 | 21.15±  1.66 | 19.82±  1.29 | 21.14±  1.66 | 20.09±  1.35 | 20.51±  1.35 | 20.45±  1.58 | 20.77±  1.36 | 21.11±  1.55 |
| trans-Geranylacetone | 3796-70-1 | 10.78±  0.97 | 10.70±  0.59 | 11.00±  0.85 | 10.96±  0.86 | 11.01±  0.82 | 10.66±  0.88 | 10.68±  0.72 | 10.58±  1.13 | 10.66±  0.93 | 10.86±  1.12 |
| Acetoin | 513-86-0 | 116.16±  8.69 | 268.04±  21.31 | 149.25±  10.92 | 123.14±  9.53 | 121.35±  9.59 | 42.73±  3.13 | 70.71±  4.88 | 74.04±  5.29 | 42.73±  2.37 | 61.25±  3.95 |
| **Others** | | | | | | | | | | | |
| α-Farnesene | 502-61-4 | 37.17±  2.15 | 33.01±  2.06 | 28.84±  3.05 | 29.16±  1.88 | 21.66±  1.77 | 23.63±  1.67 | 27.01±  1.81 | 65.48±  4.35 | 23.28±  1.68 | 58.77±  4.22 |
| Phenol | 108-95-2 | 14.28±  1.03 | 14.25±  1.22 | 17.78±  1.02 | 15.27±  1.27 | 12.66±  0.84 | 15.13±  1.20 | 16.09±  1.02 | 14.86±  1.19 | 10.56±  0.76 | 18.10±  1.13 |
| Nonanoic acid | 112-05-0 | 2.23±  0.19 | 1.98±  0.14 | 7.35±  0.38 | 7.29±  0.64 | 7.32±  0.61 | 6.18±  0.37 | 6.01±  0.33 | 3.28±  0.29 | 2.26±  0.19 | 5.09±  0.37 |

ND: Not detect.

**Supplemental Table 2** Quality analysis of each sample about RNA-seq

| Sample | Raw Reads | Raw Bases | Clean Reads | Clean Bases | Valid Bases | Q30 | GC |
| --- | --- | --- | --- | --- | --- | --- | --- |
| HF 0_1 | 49.16M | 7.37G | 48.45M | 6.86G | 93.09% | 95.30% | 47.32% |
| HF 0_2 | 43.94M | 6.59G | 43.27M | 6.13G | 92.94% | 95.29% | 47.30% |
| HF 0_3 | 49.74M | 7.46G | 49.05M | 6.99G | 93.72% | 95.34% | 47.35% |
| HF4_1 | 49.60M | 7.44G | 48.90M | 7.00G | 94.10% | 95.32% | 47.12% |
| HF 4_2 | 45.90M | 6.88G | 45.27M | 6.48G | 94.19% | 95.26% | 47.13% |
| HF 4_3 | 47.54M | 7.13G | 46.91M | 6.73G | 94.43% | 95.35% | 47.01% |
| HF 8_1 | 44.19M | 6.63G | 43.57M | 6.22G | 93.81% | 95.24% | 47.10% |
| HF 8_2 | 47.34M | 7.10G | 46.65M | 6.64G | 93.49% | 95.37% | 47.34% |
| HF 8_3 | 49.63M | 7.44G | 48.95M | 6.94G | 93.28% | 95.36% | 46.96% |
| HF 12_1 | 48.50M | 7.27G | 47.78M | 6.77G | 93.11% | 95.36% | 47.12% |
| HF 12_2 | 47.79M | 7.17G | 47.14M | 6.72G | 93.72% | 95.40% | 47.31% |
| HF 12_3 | 47.13M | 7.07G | 46.49M | 6.62G | 93.63% | 95.45% | 47.01% |
| LF 0_1 | 48.08M | 7.21G | 47.44M | 6.73G | 93.38% | 95.38% | 47.35% |
| LF 0_2 | 50.17M | 7.53G | 49.52M | 7.05G | 93.62% | 95.51% | 47.39% |
| LF 0_3 | 50.72M | 7.61G | 49.98M | 7.02G | 92.26% | 95.28% | 47.40% |
| LF 4_1 | 44.63M | 6.69G | 44.04M | 6.24G | 93.23% | 95.63% | 47.35% |
| LF 4_2 | 50.56M | 7.58G | 49.78M | 6.94G | 91.50% | 95.40% | 47.30% |
| LF 4_3 | 44.04M | 6.61G | 43.35M | 6.04G | 91.51% | 95.44% | 47.08% |
| LF 8_1 | 44.45M | 6.67G | 43.86M | 6.22G | 93.25% | 95.54% | 47.14% |
| LF 8_2 | 48.43M | 7.26G | 47.77M | 6.77G | 93.24% | 95.52% | 47.04% |
| LF 8_3 | 51.56M | 7.73G | 50.89M | 7.23G | 93.52% | 95.48% | 47.38% |
| LF 12_1 | 49.31M | 7.40G | 48.65M | 6.91G | 93.38% | 95.52% | 46.96% |
| LF 12_2 | 47.33M | 7.10G | 46.66M | 6.60G | 93.02% | 95.40% | 47.37% |
| LF 12_3 | 49.68M | 7.45G | 49.03M | 6.98G | 93.60% | 95.67% | 47.17% |

Note: HF: ‘HFBZ’ fruit, LF: ‘LFBZ’ fruit; ‘0, 4, 8, and 12’: ‘day 0, day 4, day 8, and day 12’.

**Supplemental Table 3** Analyzed expression level of volatile related genes of the FPKM values in ‘HFBZ’ and ‘LFBZ’ fruit after harvest

| Gene | ‘HFBZ’ (FPKM values) | | | |  | ‘LFBZ’ (FPKM values) | | | |
| --- | --- | --- | --- | --- | --- | --- | --- | --- | --- |
|  | 0 | 4 | 8 | 12 |  | 0 | 4 | 8 | 12 |
| *LOX1a* | 2809.116968 | 2421.293102 | 2427.221015 | 3990.776645 |  | 976.506693 | 1482.675824 | 1542.551477 | 1473.714934 |
| *LOX2a* | 1.885097091 | 15.45295723 | 14.9105165 | 11.43724681 |  | 3.435926863 | 5.641629933 | 4.204150622 | 8.304772154 |
| *LOX2b* | 2.697773345 | 0.548598915 | 0.656593168 | 1.045773431 |  | 2.792528211 | 1.751880147 | 0.696494041 | 1.698764646 |
| *LOX2c* | 606.2279596 | 249.682078 | 53.76686695 | 85.11982091 |  | 739.0499701 | 376.97221 | 229.2848517 | 95.6815802 |
| *LOX3* | 0.044911483 | 0.306472589 | 0.397681852 | 0.634987086 |  | 0.195790958 | 0.796447396 | 0.33947939 | 0.551830287 |
| *LOX5a* | 0.671623914 | 33.61865281 | 29.44211128 | 10.58330741 |  | 0.997892005 | 10.17799026 | 6.602490501 | 9.597442612 |
| *LOX7a* | 65.9304295 | 114.5339614 | 150.6568168 | 286.6974604 |  | 26.8991783 | 20.29134745 | 24.28925382 | 39.0909407 |
| *ADH* | 17.85092388 | 3.522733171 | 0.786718568 | 0.452628362 |  | 1.633318871 | 1.194514734 | 0.386731963 | 1.985773536 |
| *ADH1* | 0.227339595 | 1.339344106 | 0.986648966 | 0.912798547 |  | 0.129273631 | 0.218102107 | 0.252127987 | 0.211634181 |
| *HPL1* | 57.97756223 | 32.71570404 | 31.16848653 | 25.65650211 |  | 61.49628897 | 63.47901572 | 55.6588893 | 52.75539581 |
| *HPL2* | 13.60504183 | 10.12881442 | 4.204084838 | 4.306106586 |  | 18.21672834 | 19.29798143 | 6.88276144 | 6.373947716 |
| *AAT1* | 34.95279023 | 67.59044597 | 70.93232446 | 44.31106636 |  | 78.17942188 | 75.78515076 | 98.62388604 | 73.86975842 |

Note: ‘0, 4, 8, and 12’: ‘day 0, day 4, day 8, and day 12’.

**Supplemental Table 4** Correlation analysis of volatile components, crucial aroma related genes, and enzyme activities

|  | Total volatiles | Esters | Aldehydes | Alcohols | Ketones | *LOX1a* | *LOX2a* | *LOX5a* | *LOX7a* | *ADH1* | *AAT1* | LOX activity | ADH activity | AAT activity |
| --- | --- | --- | --- | --- | --- | --- | --- | --- | --- | --- | --- | --- | --- | --- |
| Total volatiles | 1 | 0.948^*^ | 0.930^*^ | 0.317 | 0.574 | -0.203 | 0.756 | 0.728 | -0.262 | -0.223 | 0.921^*^ | 0.939^*^ | 0.779 | 0.419 |
| Esters | 0.948^*^ | 1 | 0.785 | 0.010 | 0.409 | 0.095 | 0.830 | 0.596 | 0.050 | -0.454 | 0.851 | 0.942^*^ | 0.810 | 0.678 |
| Aldehydes | 0.930^*^ | 0.785 | 1 | 0.528 | 0.803 | -0.422 | 0.672 | 0.876 | -0.504 | -0.085 | 0.819 | 0.818 | 0.688 | 0.084 |
| Alcohols | 0.317 | 0.010 | 0.528 | 1 | 0.405 | -0.927 | -0.174 | 0.374 | -0.994^**^ | 0.765 | 0.435 | 0.161 | 0.011 | -0.635 |
| Ketones | 0.574 | 0.409 | 0.803 | 0.405 | 1 | -0.322 | 0.495 | 0.872 | -0.447 | -0.155 | 0.321 | 0.408 | 0.384 | -0.240 |
| *LOX1a* | -0.203 | 0.095 | -0.422 | -0.927 | -0.322 | 1 | 0.092 | -0.423 | 0.920^*^ | 0.763 | -0.386 | -0.183 | -0.132 | 0.728 |
| *LOX2a* | 0.756 | 0.830 | 0.672 | -0.174 | 0.495 | 0.092 | 1 | 0.773 | 0.210 | -0.744 | 0.646 | 0.874 | 0.950^*^ | 0.516 |
| *LOX5a* | 0.728 | 0.596 | 0.876 | 0.374 | 0.872 | -0.423 | 0.773 | 1 | -0.376 | -0.307 | 0.600 | 0.711 | 0.758 | -0.086 |
| *LOX7a* | -0.262 | 0.050 | -0.504 | -0.994^**^ | -0.477 | 0.920^*^ | 0.210 | -0.376 | 1 | 0.873 | -0.357 | -0.122 | 0.062 | 0.712 |
| *ADH1* | -0.223 | -0.454 | -0.085 | 0.765 | -0.155 | 0.763 | -0.744 | -0.307 | 0.873 | 1 | -0.033 | -0.383 | -0.560 | -0.644 |
| *AAT1* | 0.921^*^ | 0.851 | 0.819 | 0.435 | 0.321 | -0.386 | 0.646 | 0.600 | -0.357 | -0.033 | 1 | 0.933^*^ | 0.786 | 0.348 |
| LOX activity | 0.939^*^ | 0.942^*^ | 0.818 | 0.161 | 0.408 | -0.183 | 0.874 | 0.711 | -0.122 | -0.383 | 0.933^*^ | 1 | 0.933^*^ | 0.505 |
| ADH activity | 0.779 | 0.810 | 0.688 | 0.011 | 0.384 | -0.132 | 0.950^*^ | 0.758 | 0.062 | -0.560 | 0.786 | 0.933^*^ | 1 | 0.429 |
| AAT activity | 0.419 | 0.678 | 0.084 | -0.635 | -0.240 | -0.728 | 0.516 | -0.086 | 0.712 | -0.644 | 0.348 | 0.505 | 0.429 | 1 |

Note: Pearson correlation coefficient was used and correlations were statistically significant when * *p* < 0.05 or ** *p* < 0.01.

**Supplemental Table 5** Gene-speciﬁc primers used for RT-qPCR analysis

| Gene symbol | Genes | Primers | Sequences (5' -3') |
| --- | --- | --- | --- |
| LOC103411221 | *LOX1a* | *LOX1a*-F | TCCCAGGAGCATTCATAATACAAA |
|  |  | *LOX1a*-R | GTCTTTGGTGTATTTTTCGG |
| LOC103449741 | *LOX2a* | *LOX2a*-F | CTGTTGTGACTGTGAGGAATAAGA |
|  |  | *LOX2a*-R | TTTAGACCAATCTTTTAGCACTCC |
| LOC114825931 | *LOX2b* | *LOX2b*-F | AAAGACGACGAGGTGGTGTATGAG |
|  |  | *LOX2b*-R | AAGGTCGTCATAAGTATCATAATC |
| LOC103438366 | *LOX2C* | *LOX2C*-F | TCAAGACTATTACAGAGGGGGGAG |
|  |  | *LOX2C*-R | TCTTCGTCTCTAAACCAAGCAAAT |
| LOC103434797 | *LOX3* | *LOX3*-F | TCAGAAGAGCGTTTACGAAGTCAA |
|  |  | *LOX3*-R | TTCTAAGTCCTTTCCCATCACCTC |
| LOC103433949 | *LOX5* | *LOX5*-F | GTCAGACTTCCTTATTTATGCCCT |
|  |  | *LOX5*-R | ATGTTGACAGGGTTCACTCCA |
| LOC103433945 | *LOX7a* | *LOX7a*-F | TCTACTTGAAGACTCTCACGCTCG |
|  |  | *LOX7a*-R | CATAGTCATAGACCCTGTCCCATT |
| LOC103427164 | *HPL1* | *HPL1*-F | TAGTAAAGAGAGGTGAAACCGATT |
|  |  | *HPL1*-R | AACTAGGCAGAAAGACAGAAAACC |
| LOC114819168 | *HPL2* | *HPL2*-F | TGTTCAAGTTTCTCACTAAATGCT |
|  |  | *HPL2*-R | AACTTGGCAGAAAGACAGAAAACC |
| LOC103409569 | *ADH1* | *ADH*-F | ACTGTGGCTGTTTTCGGATTG |
|  |  | *ADH*-R | AGAACGGGACTTCGTGGGTGA |
| LOC103439766 | *ADH2* | *ADH*-F | ACTGTAAGTCGGAGGAAAGC |
|  |  | *ADH1*-R | TATGGCAATAGATGAACCCTT |
| LOC103429192 | *AAT1* | *AAT*-F | GTTTTCTTTGGCAATGTTCTCAGT |
|  |  | *AAT*-R | AACAGCAAAGTTATCCTGGTCCTC |
| XM008393049 | *Actin* | *Actin*-F | ATTCTCCTCACTGAAGCACCTCTC |
|  |  | *Actin*-R | GATTTTCATCAAGGCATCAGTCAG |
